# The ratio of ursodeoxycholyltaurine to 7‐oxolithocholyltaurine serves as a biomarker of decreased 11β‐hydroxysteroid dehydrogenase 1 activity in mouse

**DOI:** 10.1111/bph.15367

**Published:** 2021-02-04

**Authors:** Michael Weingartner, Simon Stücheli, Denise V. Kratschmar, Julia Birk, Petra Klusonova, Karen E. Chapman, Gareth G. Lavery, Alex Odermatt

**Affiliations:** ^1^ Division of Molecular and Systems Toxicology, Department of Pharmaceutical Sciences University of Basel Basel Switzerland; ^2^ Queen's Medical Research Institute University/BHF Centre for Cardiovascular Science, University of Edinburgh Edinburgh UK; ^3^ Institute of Metabolism and Systems Research University of Birmingham Birmingham UK

**Keywords:** 11β‐hydroxysteroid dehydrogenase, bile acid, biomarker, disease, glucocorticoid, inhibitor

## Abstract

**Background and Purpose:**

11β‐Hydroxysteroid dehydrogenase 1 (11β‐HSD1) regulates tissue‐specific glucocorticoid metabolism and its impaired expression and activity are associated with major diseases. Pharmacological inhibition of 11β‐HSD1 is considered a promising therapeutic strategy. This study investigated whether alternative 7‐oxo bile acid substrates of 11β‐HSD1 or the ratios to their 7‐hydroxy products can serve as biomarkers for decreased enzymatic activity.

**Experimental Approach:**

Bile acid profiles were measured by ultra‐HPLC tandem‐MS in plasma and liver tissue samples of four different mouse models with decreased 11β‐HSD1 activity: global (11KO) and liver‐specific 11β‐HSD1 knockout mice (11LKO), mice lacking hexose‐6‐phosphate dehydrogenase (*H6pd*KO) that provides cofactor NADPH for 11β‐HSD1 and mice treated with the pharmacological inhibitor carbenoxolone. Additionally, 11β‐HSD1 expression and activity were assessed in *H6pd*KO‐ and carbenoxolone‐treated mice.

**Key Results:**

The enzyme product to substrate ratios were more reliable markers of 11β‐HSD1 activity than absolute levels due to large inter‐individual variations in bile acid concentrations. The ratio of the 7β‐hydroxylated ursodeoxycholyltaurine (UDC‐Tau) to 7‐oxolithocholyltaurine (7oxoLC‐Tau) was diminished in plasma and liver tissue of all four mouse models and decreased in *H6pd*KO‐ and carbenoxolone‐treated mice with moderately reduced 11β‐HSD1 activity. The persistence of 11β‐HSD1 oxoreduction activity in the face of H6PD loss indicates the existence of an alternative NADPH source in the endoplasmic reticulum.

**Conclusions and Implications:**

The plasma UDC‐Tau/7oxo‐LC‐Tau ratio detects decreased 11β‐HSD1 oxoreduction activity in different mouse models. This ratio may be a useful biomarker of decreased 11β‐HSD1 activity in pathophysiological situations or upon pharmacological inhibition.

**LINKED ARTICLES:**

This article is part of a themed issue on Oxysterols, Lifelong Health and Therapeutics. To view the other articles in this section visit http://onlinelibrary.wiley.com/doi/10.1111/bph.v178.16/issuetoc

## Graphical abstract

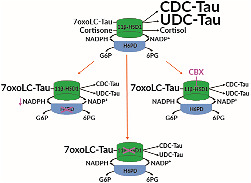

**What is already known**

- 11β‐HSD1 catalyses the oxoreduction of 11‐oxo‐glucocorticoids and 7‐oxo bile acids.
- Pharmacological inhibition of 11β‐HSD1 is considered a promising strategy to treat glucocorticoid‐dependent diseases.

**What this study adds**

- Ratio UDC‐Tau/7oxoLC‐Tau detects decreased 11β‐HSD1 activity in genetically modified mouse models and upon pharmacological inhibition.
- These ratios are better markers of decreased 11β‐HSD1 activity than concentrations of individual bile acids.

**What is the clinical significance**

- UDC‐Tau/7oxoLC‐Tau ratio provides a biomarker of the efficacy of pharmacological 11β‐HSD1 inhibition in preclinical models.

**Abbreviations**
11β‐HSD: 11β‐hydroxysteroid dehydrogenase
11KO: global 11β‐HSD1 knockout
11LKO: liver‐specific 11β‐HSD1 knockout
7oxoDCA: 7‐oxodeoxycholic acid
7oxoLCA: 7‐oxolithocholic acid
7oxoLC‐Tau: 7‐oxolithocholyltaurine
CA: cholic acid
CDCA: chenodeoxycholic acid
CDC‐Gly: chenodeoxycholylglycine
CDC‐Tau: chenodeoxycholyltaurine
CHAPS: 3‐[(3‐cholamidopropyl)dimethylammonio]‐1‐propanesulfonate
C‐Gly: cholylglycine
C‐Tau: cholyltaurine
CTRL: control
DCA: deoxycholic acid
DC‐Tau: deoxycholyltaurine
ER: endoplasmic reticulum
H6PD: hexose‐6‐phosphate dehydrogenase
*H6pd*KO: global *H6pd* knockout
HDCA: hyodeoxycholic acid
LCA: lithocholic acid
LC‐Tau: lithocholyltaurine
LLOD: lower limit of detection
MCA: muricholic acid
MOPS: 3‐(*N*‐morpholino)propanesulfonic acid
MC‐Tau: muricholyltaurine
UDCA: ursodeoxycholic acid
UDC‐Gly: ursodeoxycholylglycine
UDC‐Tau: ursodeoxycholyltaurine
UHPLC–MS/MS: ultra‐HPLC tandem‐MS

## 1 INTRODUCTION

**FIGURE 1:**
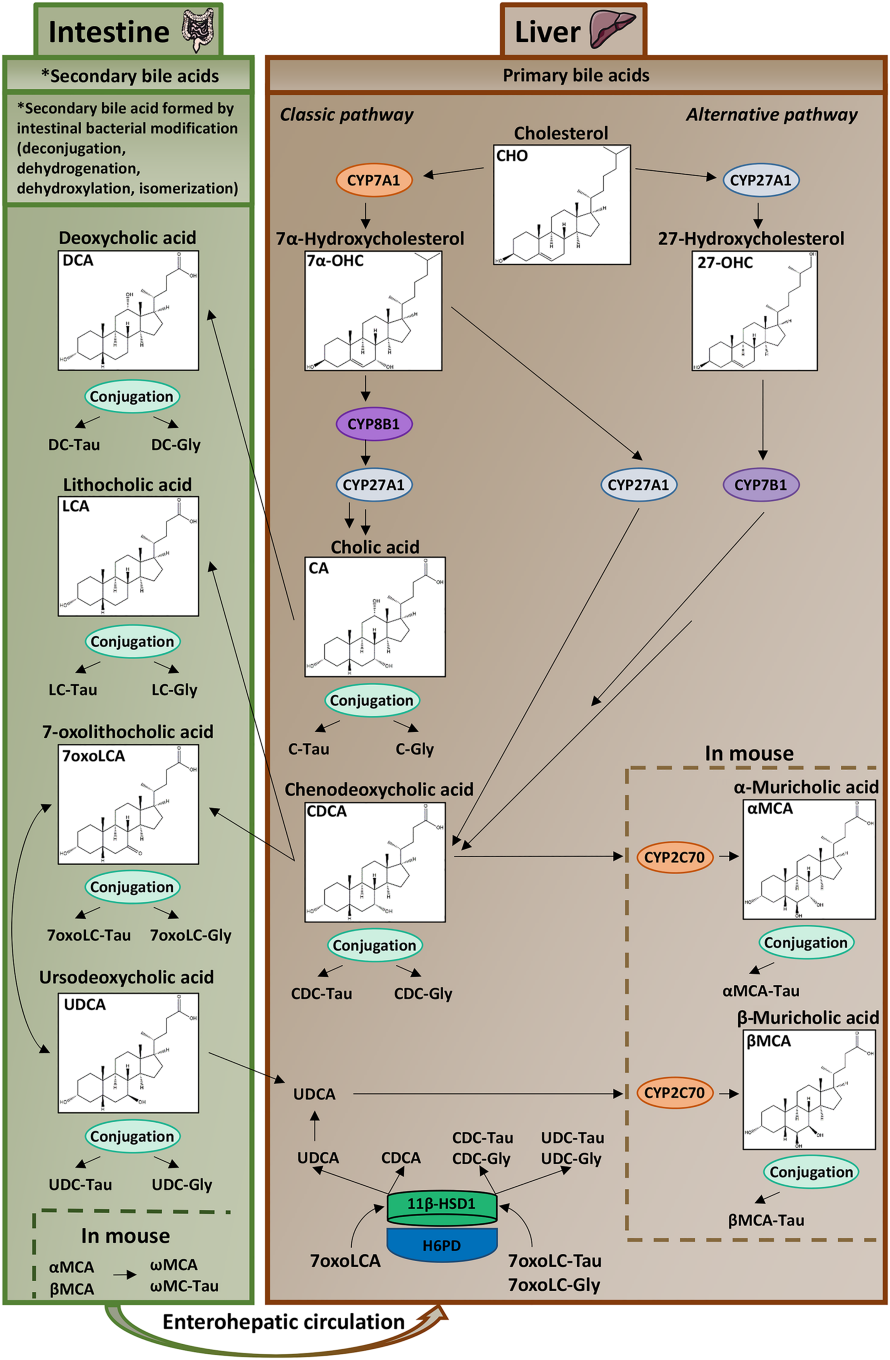
Schematic overview of bile acid homeostasis and a role for 11β‐HSD1. 11β‐HSD1 catalyses the carbonyl reduction of the substrates cortisone and 7oxoLCA to the corresponding products cortisol, and UDCA and CDCA, respectively. 11β‐HSD1 activity requires regeneration of cofactor NADPH from NADP^+^ by H6PD‐dependent conversion of glucose‐6‐phosphate (G6P) to 6‐phosphogluconate (6PG). The formation of muricholic acid metabolites by murine Cyp2c70 is indicated

A dysregulation of glucocorticoid production or a hyposensitivity or hypersensitivity to these hormones has been associated with major diseases such as osteoporosis, cognitive and mood disturbances, cardio‐metabolic disorders, cancer and immune diseases (Quax et al., 2013). Besides a tightly regulated synthesis, the tissue‐specific metabolism has a key role in mediating glucocorticoid‐regulated functions. 11β‐Hydroxysteroid dehydrogenase type 1 (11β‐HSD1) and type 2 (11β‐HSD2) catalyse the conversion of inactive 11‐oxoglucocorticoids (cortisone, 11‐dehydrocorticosterone) to potent 11β‐hydroxyglucocorticoids (cortisol, corticosterone) and the reverse reaction, respectively, and both enzymes are cell specifically expressed (Odermatt & Kratschmar, 2012). 11β‐HSD1, although catalysing both oxidation and oxoreduction *in vitro*, predominantly acts as an oxoreductase *in vivo* due to co‐expression with hexose‐6‐phosphate dehydrogenase (H6PD) that provides NADPH co‐substrate in the endoplasmic reticulum (ER) (Atanasov et al., 2004; Banhegyi et al., 2004; Lavery et al., 2006) (Figure 1). 11β‐HSD1 is essential for the therapeutic effects of pharmacologically administered cortisone and prednisone (Hult et al., 1998).

Rodent studies and clinical investigations demonstrated an association between excessive 11β‐HSD1 activity and adverse health effects including insulin resistance and type II diabetes mellitus, osteoporosis, impaired wound healing, skin aging, cognitive impairment and glaucoma (Gathercole et al., 2013; Terao & Katayama, 2016; Wyrwoll et al., 2011). Therefore, 11β‐HSD1 attracted high attention for potential therapeutic applications and a variety of small molecule inhibitors have been developed in order to assess their effects in preclinical and clinical studies (Feig et al., 2011; Freude et al., 2016; Hardy et al., 2020; Markey et al., 2017; Rosenstock et al., 2010; Schwab et al., 2017; Scott et al., 2014; Tiganescu et al., 2018; Webster et al., 2017; Ye et al., 2017).

Biomarkers of *in vivo* 11β‐HSD1 activity can facilitate preclinical and clinical investigations into states of 11β‐HSD1 deficiency and the efficacy of pharmacological inhibitors. Currently, decreased ratios of urinary (tetrahydrocorticosterone + allo‐tetrahydrocorticosterone)/tetrahydro‐11‐dehydrocorticosterone and (tetrahydrocortisol + allo‐tetrahydrocortisol)/tetrahydrocortisone are used as biomarkers for decreased 11β‐HSD1 activity in rodents and human, respectively (Abrahams et al., 2012; Courtney et al., 2008; Freude et al., 2016; Jamieson et al., 1999; Lavery et al., 2013; Webster et al., 2017). However, these ratios require analysis of urine samples (usually 24‐h sampling) and small sample volumes remain a challenge when analysing mouse urine. Moreover, the tetrahydro‐glucocorticoid ratios are strongly influenced by 11β‐HSD2 activity, as are plasma or serum cortisol/cortisone and corticosterone/11‐dehydrocorticosterone ratios (Quinkler & Stewart, 2003; Ulick et al., 1979, 1990). These glucocorticoid metabolite ratios are therefore not useful to monitor disease states with altered 11β‐HSD1 activity or to assess 11β‐HSD1 inhibitors in preclinical and clinical studies.

Besides cortisone and 11‐dehydrocorticosterone, 11β‐HSD1 can catalyse the carbonyl reduction of a broad range of substrates, including 11‐oxygenated glucocorticoids, progestins and androgens, 7‐oxygenated androgens, oxysterols and bile acids and several xenobiotics (Odermatt & Klusonova, 2015).

Experiments using human liver microsomes and HEK‐293 cells expressing human 11β‐HSD1 and H6PD revealed that human 11β‐HSD1 can convert the gut microbiota‐derived 7‐oxolithocholic acid (7oxoLCA) and its taurine‐ and glycine‐conjugated forms to chenodeoxycholic acid (CDCA) and to a lesser extent to the 7β‐stereoisomer ursodeoxycholic acid (UDCA) and their taurine‐ and glycine‐conjugated forms (Odermatt et al., 2011). Unlike human 11β‐HSD1, the mouse and rat enzymes are not stereo specific and were found to equally produce CDCA and UDCA (Arampatzis et al., 2005). A comparison of liver‐specific 11β‐HSD1 knockout (11LKO) and control (CTRL) mice showed completely abolished 7oxoLCA oxoreduction in liver microsomes from 11LKO, indicating that 11β‐HSD1 is the major if not only enzyme catalysing this reaction in the liver. Plasma and intrahepatic levels of 7oxoLCA and its taurine‐conjugated form 7‐oxolithocholyltaurine (7oxoLC‐Tau) were found to be increased in 11LKO and in global 11β‐HSD1 knockout (11KO) mice (Penno et al., 2013). Furthermore, 11KO mice exhibited increased plasma and intrahepatic levels of most bile acids, resembling a mild cholestasis phenotype.

Because we previously observed marked inter‐animal variation in circulating bile acid levels, we hypothesized that the ratios of 7β‐hydroxy‐ to 7‐oxo‐bile acids might serve as biomarkers for decreased 11β‐HSD1 activity and that such ratios may be superior markers than individual metabolite levels. We analysed plasma and liver tissue bile acids in 11KO and 11LKO mice in order to calculate the ratios of UDCA/7oxoLCA, CDCA/7oxoLCA, ursodeoxycholyltaurine (UDC‐Tau)/7oxoLC‐Tau and chenodeoxycholyltaurine (CDC‐Tau)/7oxoLC‐Tau (Penno et al., 2014; Penno, Morgan, et al., 2013). Furthermore, we analysed bile acid composition in plasma and liver tissue samples from global *H6pd* knockout (*H6pd*KO) mice as a model of decreased 11β‐HSD1 oxoreduction activity and from C57BL/6JRj mice treated with the pharmacological inhibitor carbenoxolone.

## 2 METHODS

### 2.1 Materials

Cholic acid (CA), CDCA, deoxycholic acid (DCA), lithocholic acid (LCA), UDCA, deoxycholylglycine, chenodeoxycholylglycine (CDC‐Gly), CDC‐Tau, cortisone, cortisol, corticosterone, 11‐dehydrocorticosterone, carbenoxolone, [2,2,4,4‐^2^H_4_]‐CA (>98% isotopic purity), [2,2,4,4‐^2^H_4_]‐CDCA (>98% isotopic purity) and [2,2,4,4‐^2^H_4_]‐LCA (>98% isotopic purity) were obtained from Sigma‐Aldrich (St. Louis, MO, USA). 7‐Oxodeoxycholic acid (7oxoDCA), 7oxoLCA, hyodeoxycholic acid (HDCA), α‐muricholic acid (αMCA), βMCA, ωMCA, ursodeoxycholylglycine (UDC‐Gly), lithocholyltaurine (LC‐Tau), α‐muricholyltaurine (αMC‐Tau), βMC‐Tau, ωMC‐Tau and [2,2,4,4‐^2^H_4_]‐DCA (>98% isotopic purity) were purchased from Steraloids (Newport, RI, USA). Cholylglycine (C‐Gly), cholyltaurine (C‐Tau), deoxycholyltaurine (DC‐Tau) and UDC‐Tau were obtained from Calbiochem (Läufelfingen, Switzerland). [2,2,4,4‐^2^H_4_]‐UDCA (>98% isotopic purity), [2,2,4,4‐^2^H_4_]‐C‐Gly (>98% isotopic purity), [2,2,4,4‐^2^H_4_]‐CDC‐Gly (>98% isotopic purity), [2,2,4,4‐^2^H_4_]‐UDC‐Gly (>98% isotopic purity) and [2,2,4,6,6,17α,21,21‐^2^H_8_]‐corticosterone were purchased from CDN isotopes (Pointe‐Claire, Quebec, Canada). 7oxoLC‐Tau and 7‐oxolithocholylglycine were a kind gift from Dr. Alan F. Hofmann (University of California, San Diego, CA, USA). [1,2,6,7‐^3^H]‐Cortisol was purchased from PerkinElmer (Schwerzenbach, Switzerland), [1,2‐^3^H]‐cortisone from Anawa (Kloten, Switzerland) and scintillation cocktail (IrgaSafe Plus) from Zinsser Analytic GmbH (Frankfurt am Main, Germany). Ultra‐HPLC tandem‐MS (UHPLC–MS/MS)‐grade purity methanol, acetonitrile and formic acid were obtained from Biosolve (Dieuze, France). RIPA buffer, β‐mercaptoethanol, HRP‐conjugated goat anti‐mouse secondary antibody (Cat#A0168, LOT#079M4881V, RRID:AB_257867), rabbit polyclonal anti‐H6PD antibody (Cat#HPA004824, LOT#A06407 RRID:AB_1079037), dNTPs, KAPA SYBR® FAST qPCR Kit, polyvinylidene difluoride membranes (Cat# IPVH00010, pore size: 0.45 μm), Immobilon Western Chemiluminescence HRP substrate kit and protease inhibitor cocktail were purchased from Merck (Darmstadt, Germany). Rabbit polyclonal anti‐11β‐HSD1 antibody (Cat#10004303, LOT#126826‐12, RRID:AB_10077698) was purchased from Cayman chemicals (Ann Arbor, MI, USA), HRP‐conjugated goat anti‐rabbit secondary antibody (Cat# 7074, LOT#22, RRID:AB_2099233) from Cell Signaling (Cambridge, UK) and mouse monoclonal anti‐β‐actin (ACTB) antibody (Cat#sc‐47778, LOT#D0618, Clone#C4, RRID:AB_2714189) from Santa Cruz Biotechnology (Dallas, TX, USA). Pierce® bicinchoninic acid protein assay kit and RapidOut DNA Removal kit were purchased from Thermo Fisher Scientific (Waltham, MA, USA), RNeasy Mini Kit from Qiagen (Venlo, Netherlands), GoScript Reverse Transcriptase, Oligo‐dT primers and RNasin® Ribonuclease Inhibitor from Promega (Madison, WI, USA) and primers for RT‐qPCR from Microsynth AG (Balgach, Switzerland). HEK‐293 cells (RRID:CVCL_0045) were purchased from the American Type Culture Collection (Manassas, VA, USA). FBS was obtained from Connectorate (Dietikon, Switzerland). Penicillin/streptomycin and non‐essential amino acids were purchased from BioConcept (Allschwil, Switzerland). All other reagents were purchased from Sigma‐Aldrich.

### 2.2 Animal experimentation

Animal studies are reported in compliance with the ARRIVE guidelines (Percie du Sert et al., 2020) and with the recommendations made by the *British Journal of Pharmacology* (Lilley et al., 2020). 11KO mice were described earlier (Semjonous et al., 2011); a targeted deletion of exon 5 in *Hsd11b1* was obtained using the Cre‐loxP system. E14TG2a embryonic stem cells bearing a triloxed allele were injected into C57BL/6J (RRID:IMSR_JAX:000664) blastocysts and chimeric mice were mated with C57BL/6J females. Mice heterozygous for a triloxed allele were crossed with ZP3‐Cre to create the null allele and bread to homozygosity to generate 11KO. 11KO were intercrossed to maintain the C57BL/6J/129SvJ background. To obtain 11LKO, the floxed homozygous 11KO mice on the mixed C57BL/6J/129SvJ background were crossed with Albumin‐Cre transgenic mice on a C57BL/6J background to target Cre expression to hepatocytes (Lavery et al., 2012). Mice were group housed at the University of Birmingham (Birmingham, UK), in a climate‐controlled facility under standard conditions on a 12‐h light/dark cycle and fed ad libitum with standard chow (Cat#D12328, Research Diets, Inc., New Brunswick, USA) and free access to drinking water. 11KO, 11LKO and their respective control littermates, 15‐week‐old male mice, were fasted overnight and anaesthetized with isoflurane prior to collection of blood samples by intra‐cardiac puncture and isolation of livers. Samples were collected between 7:00 and 10:00. Studies with 11KO and 11LKO were conducted under Home Office license PPL 70/8516, following approval by the Joint Ethics and Research Governance Committee of the University of Birmingham in accordance with the United Kingdom Animals (Scientific Procedures) Act, 1986 and the EU Directive 2010/63/EU for animal experiments.

For *H6pd*KO mice, a deletion of exons 2 and 3 in *H6pd* was generated by homologous recombination in 129SvJ embryonic stem cells, followed by injection into C57BL/6J blastocysts and resulting chimeric mice were mated with C57BL/6J female mice. Mice were intercrossed to maintain the C57BL/6J/129SvJ background. From these, heterozygous mice were intercrossed to obtain *H6pd*KO mice and control littermates (*H6pd* ^tm1Pmst^, RRID:MGI:3624665, Lavery et al., 2006). *H6pd*KO mice were transferred from the University of Birmingham to the PharmaCenter animal facility (University of Basel, Switzerland), where they were bred and housed. The C57BL/6JRj (RRID:MGI:2670020) mice used for carbenoxolone treatment were purchased from Janvier Laboratories (Saint Berthevin, France). These animals were acclimatized to the new environment (Basel, Switzerland) for 1 week prior to the experiment. Experiments with *H6pd*KO and C57BL/6JRj mice were performed on 10‐ to 12‐week‐old males. The Cantonal Veterinary Office in Basel, Switzerland, approved all procedures (cantonal licenses 2758_26280 and 2758_29462). Mice were group housed in acclimate‐controlled facility under standard conditions and a 12‐h light/12‐h dark cycle with free access to standard chow (Cat#3432, KLIBA NAFAG, Kaiseraugst, Switzerland) and drinking water in ventilated cages. All experiments were performed between 7:00 and 10:00. Mice (not fasted) were killed by exposure to CO_2_ until respiratory arrest was observed, absence of pain reaction was verified and cardiac puncture was performed immediately to collect blood. Plasma samples were prepared by centrifugation at 2,000× *g*, 10 min, 4°C and stored at −80°C. Liver tissue samples were either immediately used for 11β‐HSD1 activity assay or snap frozen in liquid nitrogen and stored at −80°C until further analysis. In experiments including carbenoxolone treatment, C57BL/6JRj mice received carbenoxolone (100 mg·kg^−1^·day^−1^) for 4 days via i.p. injection (50 mg·kg^−1^ in PBS at 7:00 and 17:00). The dose was established in preliminary experiments. Animals for the carbenoxolone treatment were distributed, treated and killed in randomized block design (study performed at the beginning of 2017). Only the person handling the animals was aware of the group allocation. Animals which displayed obvious signs of health issues like excessive loss of weight (more than 20%) were excluded from the study. These criteria were defined prior to the study. *H6pd*KO and corresponding control littermates were randomly assigned to groups. Resulting sample material from plasma and liver tissue (experimental unit) was extracted, measured and analysed in a blinded and simple randomized design. Final sample batches from UHPLC–MS/MS analysis were transferred to Excel and unblinded for statistical evaluations.

### 2.3 Quantification of bile acids and steroids

Stock solutions of analytes, internal standards (10 mmol·L^−1^) and mixtures of analytes (Penno et al., 2013) were prepared in methanol. Calibrators for plasma analysis of bile acids (25 μl) or steroids (50 μl) were prepared by serial dilution of charcoal‐treated mouse plasma spiked with analytes. Calibration curves for liver samples were prepared by serial dilution of analytes in PBS (200 μl, pH 7.2). Calibration curves for cell culture supernatant were prepared by serial dilution in serum‐free culture medium. All calibrators were subsequently treated as samples. An internal standard mixture was prepared of ^2^H_4_‐CA, ^2^H_4_‐CDCA, ^2^H_4_‐DCA, ^2^H_4_‐UDCA, ^2^H_4_‐C‐Gly, ^2^H_4_‐CDC‐Gly, ^2^H_4_‐UDC‐Gly and ^2^H_4_‐LCA for quantification of bile acids in plasma and liver, of ^2^H_4_‐CDCA, ^2^H_4_‐UDCA and ^2^H_4_‐LCA for bile acid quantification in cell culture supernatant, and of ^2^H_8_‐corticosterone and ^2^H_4_‐cortisone for steroid quantification.

Plasma samples (25 μl) for bile acids were diluted with water (75 μl), spiked with internal standard (100 nmol·L^−1^) and subjected to protein precipitation by isopropanol (900 μl). Samples were incubated (30 min, 4°C, 1,400 rpm) and centrifuged (10 min, 4°C, 16,000× *g*), and supernatants were transferred into a fresh tube. Liver samples (approximately 30 mg) were homogenized using a Precellys 24 tissue homogenizer (Bertin Instruments, Montigny‐le‐Bretonneux, France) (4°C, 3×, 30 s at 6,500 rpm, cycle break 30 s) in water–chloroform–methanol (1 ml, 20/20/60; v/v/v) containing internal standard (100 nmol·L^−1^). Samples were incubated (15 min, 850 rpm, 37°C) and centrifuged (10 min, 25°C, 16,000× *g*), and supernatants (800 μl) were transferred to a fresh tube. All plasma and liver samples were re‐extracted and supernatants combined. Cell culture supernatant (450 μl) was spiked with internal standard (100 nmol·L^−1^), subjected to protein precipitation by isopropanol (1 ml), incubated (30 min, 4°C, 1,300 rpm) and centrifuged (10 min, 4°C, 16,000× *g*), and the resulting supernatant was transferred to a fresh tube. Supernatants of plasma (2 ml), liver (1.6 ml), and cell culture (1.45 ml) were evaporated to dryness using a Genevac EZ‐2 evaporator (35°C). Plasma samples (50 μl) for steroids were spiked with internal standard (3.3 nmol·L^−1^) and extracted by solid phase extraction (3 cc, Oasis HLB cartridges) as described (Strajhar et al., 2016). Extracts (1 ml) were evaporated to dryness (35°C). Sample residues for bile acid detection were reconstituted (10 min, 25°C, 1,300 rpm) in methanol–water (50/50 v/v; 50 μl for plasma, 200 μl for liver, 50 μl for cell culture supernatant). Sample residues for steroid measurement were reconstituted (10 min, 4°C, 1,300 rpm) in methanol (25 μl). All reconstituted samples were sonicated (10 min, 25°C) and centrifuged (10 min, 25°C, 16,000× *g*), and supernatants were transferred to glass vials.

The injection volume for bile acid detection was 2 μl (plasma and cell culture supernatant) or 3 μl (liver) and for plasma steroids 5 μl. Samples were stored at −20°C until analysis by UHPLC–MS/MS as described earlier (Penno, Arsenijevic, et al., 2013) with minor modifications. Briefly, analytes were detected by multiple reaction monitoring using an Agilent Triple Quadrupole 6490 instrument with electrospray ionization and polarity switching. Analytes were separated with a reverse‐phase column (Acquity UPLC BEH C18, 1.7 μm, 2.1 × 150 mm, Waters, Milford, MA, USA) at 65°C within 17.5 min for bile acids or 10 min for steroids. The mobile phase consisted of water–acetonitrile–formic acid (A) (95/5/0.1, v/v/v) and (B) (5/95/0.1, v/v/v). Gradient elution (% mobile phase B) was performed at constant flow (0.63 ml·min^−1^): bile acids, 0–8 min (25%); 8–17.5 min (35–68.25%); 17.5–18 min (68.25–25%); followed by a washout 18–20 min (25–100%) and 20–22 min (100%); and steroids, 0–10 min (25–70%); followed by a washout 10–12 min (100%). The column was post run reconstituted to initial %B within 2 min prior to further injections. Data acquisition and quantification were performed using MassHunter (Acquisition software version B.09.00, build 9.0.9037.0 and quantitative software version B.07.01, build 7.1.524.0).

### 2.4 Expression of mouse 11β‐HSD1 in HEK‐293 cells and oxoreduction of 7oxoLCA

HEK‐293 cells, cultured in DMEM supplemented with 10% FBS, 10 mmol·L^−1^ HEPES, 100 units·ml^−1^ penicillin, 0.1 mg·ml^−1^ streptomycin and non‐essential amino acids in a 5% CO_2_ atmosphere at 37°C, were transfected with plasmid expressing mouse 11β‐HSD1 bearing a C‐terminal FLAG epitope (Arampatzis et al., 2005) using the calcium phosphate transfection method. The medium was changed 8 h after transfection and after 48 h, cells were cultured in medium containing G‐418 as selection antibiotic (800 μg·ml^−1^). HEK‐293 cells stably expressing mouse 11β‐HSD1 are referred to as MO1F cells. Cells (100,000 per well) were seeded on poly‐l‐lysine coated 24‐well plates. After 24 h, cells were washed twice with serum‐free culture medium and incubated with 400 nmol·L^−1^ 7oxoLCA, either with or without 5 μmol·L^−1^ carbenoxolone, for 0, 4 and 24 h. Of the supernatant, 450 μl was then transferred to a 2‐ml tube and stored at −20°C until bile acid quantification.

### 2.5 *In vivo* 11β‐HSD1 activity assessment

Mice were injected i.p. with 5 mg·kg^−1^ of cortisone (in DMSO). After 10 min, mice were killed by CO_2_ asphyxiation and cardiac puncture was performed immediately to collect blood. Plasma was prepared and stored as described above. Plasma was extracted, and cortisone and cortisol levels were measured by UHPLC–MS/MS as described above.

### 2.6 *Ex vivo* activity assay

Freshly isolated liver tissue samples (50–100 mg) were placed in tubes, followed by injection of radiolabelled substrate mixture (10 μl containing either 950 nmol·L^−1^ cortisone + 50 nmol·L^−1 3^H‐cortisone [60 Ci·mmol^−1^] or 950 nmol·L^−1^ cortisol + 50 nmol·L^−1^ of ^3^H‐cortisol [70 Ci·mmol^−1^]). Samples were incubated at 16°C for 10 min. Freshly isolated epididymal white adipose tissue samples (50–100 mg) were similarly treated but incubated for 10 min at 37°C. Reactions were terminated by snap freezing and stored at −80°C. For the extraction of cortisone and cortisol, samples were sonicated for 30 s in 200‐μl water; 750‐μl ethyl acetate was added, followed by incubation (15 min, 4°C, 1,300 rpm) then centrifugation (10 min, 4°C, 16,100× *g*). The supernatant (600 μl) was transferred to a fresh tube and the extraction repeated. Combined supernatants (1.2 ml) were evaporated to dryness in a Genevac EZ‐2 (35°C). Residues were reconstituted in 1.2‐ml methanol by sonication (10 min, 25°C), followed by evaporation to dryness and storage at −80°C. To the white adipose tissue samples, 1‐ml methanol was added, samples were vortexed for 10 s and centrifuged (10 min, 25°C, 21,000× *g*), and supernatant (950 μl) was transferred to a new tube. Samples were evaporated to dryness and stored at −80°C. Residues of the liver and white adipose tissue samples were then reconstituted in 40‐μl methanol containing 0.5 mmol·L^−1^ of cortisone and cortisol, by sonication (10 min, 25°C). Samples (10 μl) were then separated on SIL G‐25 UV TLC plates (Macherey‐Nagel, Düren, Germany) in chloroform/methanol (90/10, v/v) and analysed by scintillation counting.

### 2.7 Quantification of mRNA expression by RT‐qPCR

Liver samples (approximately 14 mg) were homogenized (6,500 rpm, 4°C, 30 s; Precellys 24 tissue homogenizer in 400‐μl RLT buffer [RNeasy Mini, Qiagen]) and centrifuged (3 min, 25°C, 16,000× *g*). Total RNA was isolated from the supernatant (QIAcube, standard protocol for animal tissues and cells, Qiagen) and genomic DNA was removed by DNAse digestion. RNA was quantified and transcribed (500 ng) into cDNA and then qPCR was performed (4‐ng cDNA per reaction in triplicate, 40 cycles) using KAPA SYBR® FAST. Oligonucleotide primers: *Hsd11b1* forward 5′‐ TGG TGC TCT TCC TGG CCT‐3′, reverse 5′‐ CCC AGT GAC AAT CAC TTT CTT T‐3′; *H6pd* forward 5′‐ CTT GAA GGA GAC CAT AGA TGC G‐3′, reverse 5′‐ TGA TGT TGA GAG GCA GTT CC‐3′; peptidylpropyl isomerase A (Ppia) forward 5′‐ CAA ATG CTG GAC CAA ACA CAA ACG‐3′, reverse 5′‐ GTT CAT GCC TTC TTT CAC CTT CCC‐3′. Comparison of gene expression was performed using the 2‐ΔCT method with *Ppia* as the internal control (Schmittgen & Livak, 2008).

### 2.8 Protein expression analysis by Western blot

Liver samples (approximately 6 mg) were homogenized (6,500 rpm, 30 s, 4°C, Precellys 24 tissue homogenizer) in RIPA buffer (450 μl) containing protease inhibitor cocktail and centrifuged (4 min, 4°C, 16,000× *g*). Protein concentration was quantified by using a bicinchoninic acid protein assay and samples were prepared (5 min at 95°C) in Laemmli solubilization buffer (60 mmol·L^−1^ Tris–HCl, 10% glycerol, 0.01% bromophenol blue, 2% sodium dodecyl sulfate, pH 6.8, 5% β‐mercaptoethanol). The protein extract (20 μg) was separated by 14% SDS‐PAGE and transferred to polyvinylidene difluoride membranes. The membranes were blocked (1 h, 25°C) in TBS‐T (5% defatted milk, 20 mmol·L^−1^ Tris buffered saline with 0.1% Tween‐20). All antibody dilutions and incubations were performed in TBS‐T. 11β‐HSD1 protein expression was measured with rabbit polyclonal anti‐11β‐HSD1 antibody (1:1,000, 4°C, overnight). The membrane was washed and incubated with HRP‐conjugated goat anti‐rabbit secondary antibody (1:2,000, 25°C, 1 h). H6PD protein expression was determined using rabbit polyclonal anti‐H6PD antibody (1:1,000, 4°C, overnight) and HRP‐conjugated goat anti‐rabbit secondary antibody (1:2,000, 25°C, 1 h). ACTB was detected using mouse monoclonal anti‐ACTB antibody (1:1,000, 4°C, overnight) followed by HRP‐conjugated goat anti‐mouse secondary antibody (1:4,000, 25°C, 1 h). Protein content was visualized by Immobilon Western Chemiluminescence HRP substrate kit. 11β‐HSD1 and H6PD were quantified by densitometry normalized to ACTB protein levels using ImageJ software (version 1.53n, RRID:SCR_003070). The Immuno‐related procedures used comply with the recommendations made by the *British Journal of Pharmacology* (Alexander et al., 2018).

### 2.9 Estimation of NADPH levels in the ER

A method by Rogoff et al. (2010) to estimate NADPH content in liver microsomes was modified. Approximately 100 mg of frozen mouse liver tissue was thawed and homogenized in nine volumes of buffer containing 50 mmol·L^−1^ KCl, 2 mmol·L^−1^ MgCl_2_, 0.25 mol·L^−1^ sucrose, 20 mmol·L^−1^ Tris, pH 7.5 and protease inhibitor cocktail. The homogenate was centrifuged at 12,000× *g* at 4°C for 20 min and the supernatant was centrifuged again at 105,000× *g* at 4°C for 60 min. The resulting pellet was resuspended in 600 μl homogenization buffer and centrifuged again at 105,000× *g* at 4°C for 60 min. The pellet was then resuspended in 50‐μl buffer containing 100 mmol·L^−1^ KCl, 20 mmol·L^−1^ NaCl, 1 mmol·L^−1^ MgCl_2_, 20 mmol·L^−1^ 3‐(*N*‐morpholino)propanesulfonic acid (MOPS), pH 7.2 and protease inhibitor cocktail. Protein concentration was determined by using a bicinchoninic acid assay. Resuspended microsomes (protein concentration: 0.5 mg·ml^−1^) were permeabilized with alamethicin (0.1 mg·mg^−1^ protein) and 3‐[(3‐cholamidopropyl)dimethylammonio]‐1‐propanesulfonate (CHAPS) 0.25% (w/v) and EDTA (final concentration of 160 nmol·L^−1^) were added. Permeabilized microsomes were incubated for 30 min at 4°C with mixing every 5 min. Next, 100 μl of microsomal preparation per well were incubated in a 96‐well plate at 25°C for 5 min. The absorbance of total reduced pyridine nucleotides ([NADPH] + [NADH]) was measured at wavelength 335–345 nm. NADPH levels were then determined by incubating 100 μl of permeabilized microsomes with 1.4 IU GSH reductase and 0.75 mmol·L^−1^ of oxidized GSH for 20 min at 25°C whilst shaking at 350 rpm. The resulting decrease in absorbance is a measure of the NADPH content. Absorbance of total reduced pyridine nucleotides was measured against buffer in the absence of microsomes. To determine NADPH content, the absorbance of buffer containing 1.4 IU and 0.75 mmol·L^−1^ GSSG was subtracted from the absorbance obtained from the microsomal preparation. All samples were tested at least in duplicate.

### 2.10 Statistical analysis

Sample size ranged from seven to 20 animals. The different sample sizes for the different parameters measured were chosen according to experiments from previous studies where statistically significant effects have been observed. Data were tested for normal distribution by a D'Agostino and Pearson omnibus normality test followed by a non‐parametric (two‐tailed) Mann–Whitney *U* test for analysis of significance. Data represent mean ± SEM. All statistical analyses were performed using GraphPad Prism 5.0 software (RRID:SCR_002798), and *P* < 0.05 was considered significant. Effect sizes were determined for bile acid concentrations measured by UHPLC–MS/MS in plasma and liver samples from transgene or treated mice versus their respective controls (Table S1). Data represent the effect size calculated based on Cohen's *d* effect size (ES *d*) with correction of unequal sample sizes for the analysis of data from non‐parametric analysis (Mann–Whitney *U* test) calculated as Hedges' *g* with corresponding confidence intervals of the effect size. The data and statistical analysis comply with the recommendations of the *British Journal of Pharmacology* on experimental design and analysis in pharmacology (Curtis et al., 2018).

### 2.11 Nomenclature of targets and ligands

Key protein targets and ligands in this article are hyperlinked to corresponding entries in the IUPHAR/BPS Guide to PHARMACOLOGY http://www.guidetopharmacology.org and are permanently archived in the Concise Guide to PHARMACOLOGY 2019/20 (Alexander et al., 2019).

## 3 RESULTS

### 3.1 Increased circulating bile acids in 11KO mice

A previous study indicated that 11β‐HSD1 is the only enzyme catalysing the conversion of 7oxoLCA to CDCA and UDCA, reporting an accumulation of 7oxoLCA and 7oxoLC‐Tau in plasma and liver tissue from 11KO mice, although with large inter‐individual variations (Penno, Morgan, et al., 2013). Here, we have performed a reanalysis of the bile acid profiles from the previous study which shows increased levels of almost all bile acids (primary and secondary, free and conjugated) in plasma samples from 11KO mice, compared to control littermates, resembling a mild cholestasis phenotype (Table 1). However, due to the high inter‐individual variations, only some values reached significance. The levels of taurine‐conjugated bile acids were almost an order of magnitude higher than those of their free forms. Because glycine‐conjugated bile acids are considered to be of minor importance or absent in mice, consistent with previous findings (Alnouti et al., 2008; Garcia‐Canaveras et al., 2012; Penno et al., 2014), they were not included in the analysis here. In liver tissue of 11KO, free bile acids were elevated or tended to be elevated, with the exception of the 7β‐hydroxylated bile acid βMCA that was fourfold lower. The taurine‐conjugated bile acids showed a weak trend to be increased in livers of 11KO. However, 7oxoLC‐Tau was more than 10‐fold higher in livers of 11KO mice compared to control littermates (CTRL) and UDC‐Tau was about 30‐fold lower.

**TABLE 1:** Bile acid profiles in plasma and liver of 11KO mice

Analyte	Plasma (nmol·L^−1^)		Liver (fmol·mg^−1^)	
	CTRL	11KO	CTRL	11KO
	(*n* = 18)	(*n* = 17)	(*n* = 9)	(*n* = 9)
CA	703 ± 323	18,836 ± 10,398	1,673 ± 558	6,911 ± 4,710
CDCA	13.9 ± 3.8	192 ± 108	38 ± 10	49 ± 16
DCA	289 ± 71	1,603 ± 687	239 ± 53	318 ± 88
7oxoDCA	908 ± 439	29,886 ± 14,966*	1,396 ± 455	13,196 ± 9,162
HDCA	17.7 ± 4.0	236 ± 70*	74 ± 15	343 ± 124*
αMCA	101 ± 48	4,578 ± 2,091*	340 ± 90	4,018 ± 2,136*
βMCA	572 ± 165	826 ± 355	3,457 ± 968	897 ± 387*
ωMCA	604 ± 190	2,351 ± 1,182	1,461 ± 450	1,506 ± 644
UDCA	69 ± 18	486 ± 223	168 ± 38	291 ± 184
7oxoLCA	10.2 ± 3.1	201 ± 100*	18.2 ± 4.4	67 ± 24
αMCA/βMCA	0.22 ± 0.05	9.1 ± 5.0*	0.12 ± 0.21	3.65 ± 0.60*
UDCA/7oxoLCA	41 ± 18	16.9 ± 13.8*	13.7 ± 4.2	4.5 ± 1.2
CDCA/7oxoLCA	4.6 ± 2.4	1.93 ± 0.98	2.3 ± 0.5	1.03 ± 0.14*
C‐Tau	3,578 ± 2,331	110,304 ± 94,731	32,352 ± 7,659	51,276 ± 15,249
CDC‐Tau	266 ± 175	3,961 ± 3,339	991 ± 185	1,980 ± 765
DC‐Tau	1,412 ± 863	12,212 ± 9,988	5,760 ± 1,324	7,711 ± 2,315
LC‐Tau	6.3 ± 4.5	89 ± 69	59 ± 11	85 ± 21
αMCA‐Tau + βMCA‐Tau	4,203 ± 2,384	29,184 ± 23,757	19,635 ± 4,314	20,556 ± 7,700
ωMCA‐Tau	5,132 ± 2,836	17,184 ± 13,056	24,404 ± 4,625	14,609 ± 4,313
UDC‐Tau	331 ± 185	2,947 ± 2,920	1,673 ± 350	53 ± 27*
7oxoLC‐Tau	143 ± 77	5,931 ± 3,962	174 ± 34	2,718 ± 1,135*
UDC‐Tau/7oxoLC‐Tau	2.2 ± 0.3	0.30 ± 0.11*	9.5 ± 1.6	0.020 ± 0.004*
CDC‐Tau/7oxoLC‐Tau	1.05 ± 0.23	0.63 ± 0.09	6.9 ± 1.1	0.83 ± 0.06*
Sum primary BA	1,458 ± 493.0	23,782 ± 12,339	5,675 ± 1,570	12,167 ± 7,413
Sum primary BA‐Tau	8,379 ± 4,391	137,957 ± 117,634	54,651 ± 12,386	73,866 ± 23,548

*Note*: The results represent mean ± SEM (nmol·L^−1^ and fmol·mg^−1^ for plasma and liver, respectively). Analyte concentrations with a S/N ≤ 3 represent the LLOD of the UHPLC–MS/MS method. Samples yielding a concentration below LLOD were included as LLOD/2 (nmol·L^−1^ and fmol·mg^−1^, respectively) in the calculations of a specific analyte. Blue‐ and yellow‐coloured boxes indicate statistically significant increases and decreases, respectively. Unequal group sizes reflect exclusion of one plasma sample due to insufficient collection of blood sample volume and the availability of only nine livers due to the use of nine randomly assigned livers for gene expression analyses in a previous study.

Abbreviations: 11KO, global *Hsd11b1* knockout; CTRL, control littermates.

* *P* < 0.05 significantly different as indicated; non‐parametric, Mann–Whitney *U* test (two‐tailed).

### 3.2 The UDC‐Tau/7oxoLC‐Tau ratio in plasma and liver tissue detects the lack of 11β‐HSD1 activity in 11KO mice

**FIGURE 2:**
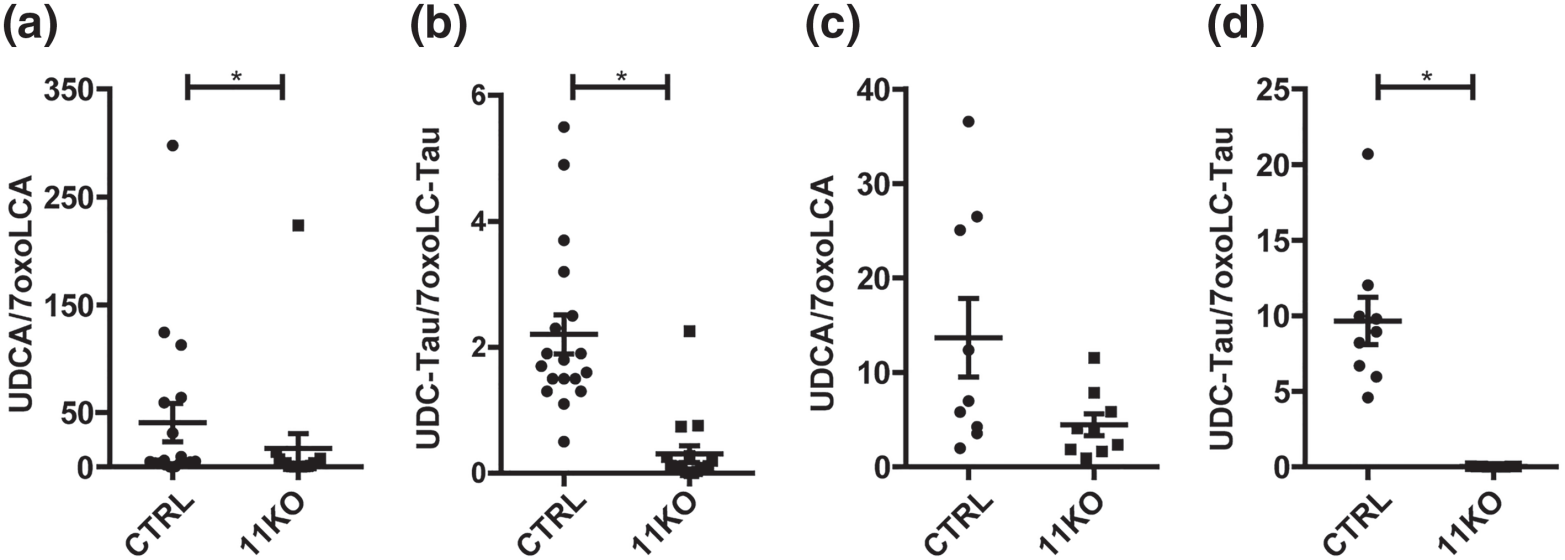
Plasma and liver tissue UDCA/7oxoLCA and UDC‐Tau/7oxoLC‐Tau ratios in 11KO mice. (a) UDCA/7oxoLCA ratios and (b) UDC‐Tau/7oxoLC‐Tau ratios in plasma of 11KO mice (CTRL *n* = 18; 11KO *n* = 17); (c) UDCA/7oxoLCA ratios and d) UDC‐Tau/7oxoLC‐Tau ratios in liver tissue of 11KO mice (CTRL *n* = 9; 11KO *n* = 9). Analyte concentrations defined by a S/N ≤ 3 represent the LLOD of the UHPLC–MS/MS method. Samples yielding a concentration below LLOD were included as LLOD/2 (nmol·L^−1^ and fmol·mg^−1^ for plasma and liver, respectively) in the calculations of a specific analyte. The results represent mean ± SEM. *P < 0.05 significantly different as indicated; non‐parametric, Mann–Whitney *U* test (two‐tailed). Unequal group sizes reflect exclusion of one plasma sample due to insufficient collection of blood sample volume and the availability of only nine livers due to the use of nine randomly assigned livers for gene expression analysis in a previous study

The concentrations of the 11β‐HSD1 substrates 7oxoLCA and 7oxoLC‐Tau increased about 20‐fold and 40‐fold, respectively, in plasma of 11KO compared to CTRL (Table 1, Figure S1), with large inter‐individual variations, as reported earlier (Penno, Morgan, et al., 2013). It needs to be noted that no outliers were excluded from the analysis. The respective products of 11β‐HSD1, that is, CDCA, UDCA and their taurine‐conjugated forms, also were higher in 11KO plasma compared to CTRL, although clearly less pronounced than the 7‐oxo metabolites. In liver tissue, 7oxoLCA was 3.7‐fold and 7oxoLC‐Tau 15‐fold higher in 11KO compared to CTRL (Table 1, Figure S2). The respective products CDCA, UDCA and CDC‐Tau tended to increase, whereas UDC‐Tau decreased 30‐fold. Importantly, the 11β‐HSD1 product to substrate ratios (the ratio of CDCA and UDCA and their taurine‐conjugated forms to the respective 7oxo metabolites) showed less variation than the individual metabolite concentrations (Figures S1 and S2). UDC‐Tau/7oxoLC‐Tau was the most distinguishing marker for the lack of 11β‐HSD1 activity when considering both plasma and liver tissue samples (Table 1, Figure 2).

Interestingly, in plasma and liver tissue of 11KO mice, the levels of the 7α‐hydroxylated bile acid αMCA were 45‐fold and 12‐fold higher than in CTRL, whereas its 7β‐hydroxylated form βMCA was not different in CTRL plasma but 3.8‐fold lower in liver tissue (Table 1). The respective αMCA/βMCA ratios were 40‐fold and 30‐fold higher in 11KO compared to CTRL, suggesting a possible effect of 11β‐HSD1 on isomerization. Due to a limitation of the applied analytical method, αMC‐Tau and βMC‐Tau could not be separated and therefore, the corresponding ratio not determined.

### 3.3 11LKO mice exhibit decreased plasma and liver tissue UDC‐Tau/7oxoLC‐Tau ratios

The liver shows the highest 11β‐HSD1 expression; nevertheless, an earlier study reported 25–30% residual *in vivo* (whole body) 11β‐HSD1 oxoreduction activity in 11LKO mice lacking 11β‐HSD1 specifically in hepatocytes (Lavery et al., 2006). Thus, 11LKO mice represent a model of reduced 11β‐HSD1 activity but with complete loss of activity in hepatocytes.

**FIGURE 3:**
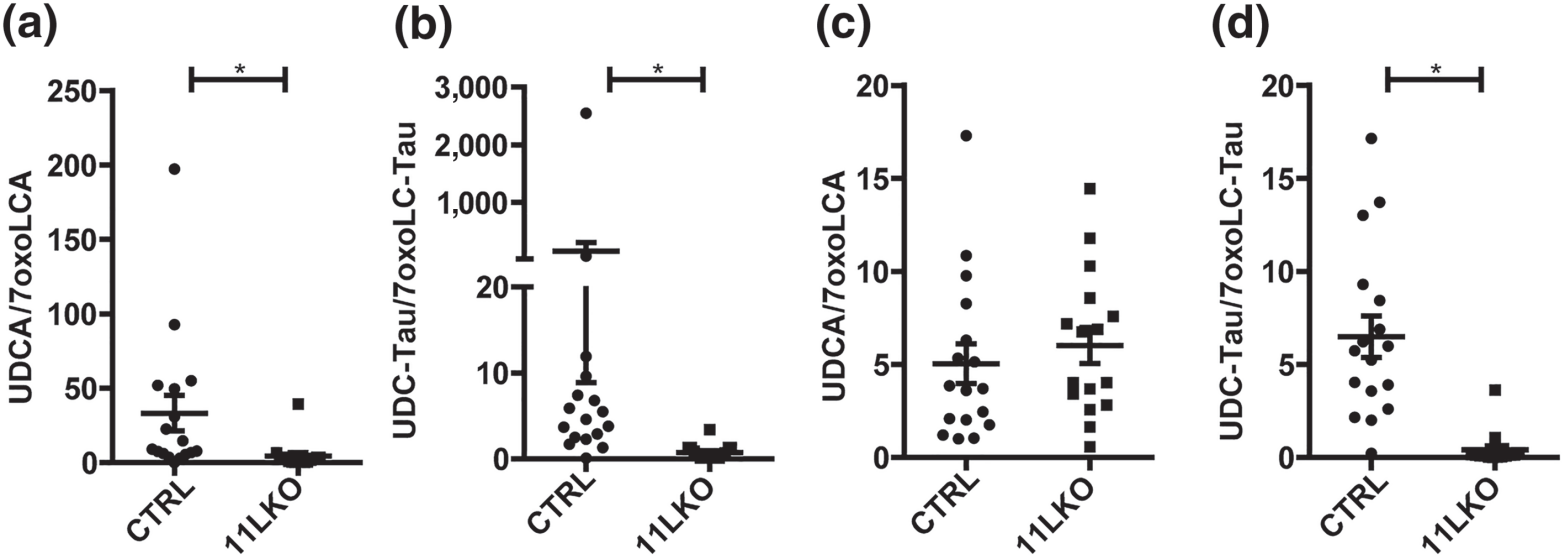
Plasma and liver tissue UDCA/7oxoLCA and UDC‐Tau/7oxoLC‐Tau ratios in 11LKO mice. (a) UDCA/7oxoLCA ratios and (b) UDC‐Tau/7oxoLC‐Tau ratios in plasma of 11LKO mice (CTRL *n* = 17; 11LKO *n* = 16); (c) UDCA/7oxoLCA ratios and (d) UDC‐Tau/7oxoLC‐Tau ratios in liver tissue of 11LKO mice (CTRL *n* = 17; 11LKO *n* = 16). Analyte concentrations defined by a S/N ≤ 3 represent the LLOD of the UHPLC–MS/MS method. Samples yielding a concentration below LLOD were included as LLOD/2 (nmol·L^−1^ and fmol·mg^−1^ for plasma and liver, respectively) in the calculations of a specific analyte. The results represent mean ± SEM. *P < 0.05 significantly different as indicated; non‐parametric, Mann–Whitney *U* test (two‐tailed). Unequal group sizes reflect exclusion of one 11LKO animal due to unexpected health issues prior to reaching the age for the experiment

The free bile acids in plasma and liver tissue of 11LKO tended to be higher compared to CTRL (Table 2), an effect considerably more pronounced in 11KO (Table 1). 7oxoLCA was 11‐fold higher in plasma and twofold in liver tissue (Table 2). The plasma UDCA/7oxoLCA and CDCA/7oxoLCA ratios were sevenfold and twofold lower in 11LKO compared to CTRL, whilst remaining unchanged in liver tissue. Plasma 7oxoLC‐Tau was slightly more abundant than its free form and it was 20‐fold higher in 11LKO than in CTRL, whilst CDC‐Tau was not different, and UDC‐Tau was sixfold lower in 11LKO, resulting in significantly decreased product to substrate ratios (Table 2, Figure 3; see also Figure S3 for individual data points). In liver tissue, 7oxoLC‐Tau was 5.5‐fold increased, CDC‐Tau not different and UDC‐Tau threefold lower in 11LKO compared to CTRL (Table 2; see also Figure S4). In agreement with 11KO, the CDC‐Tau/7oxoLC‐Tau and UDC‐Tau/7oxoLC‐Tau ratios were lower in 11LKO liver tissue compared to CTRL (fivefold and 16‐fold, respectively). The αMCA/βMCA ratio was 3.3‐fold higher in plasma and 7.3‐fold in liver tissue of 11LKO (Table 2).

**TABLE 2:** Bile acid profiles in plasma and liver of 11LKO mice

Analyte	Plasma (nmol·L^−1^)		Liver (fmol·mg^−1^)	
	CTRL	11LKO	CTRL	11LKO
	(*n* = 17)	(*n* = 16)	(*n* = 17)	(*n* = 16)
CA	528 ± 87	3,141 ± 1,837	2,004 ± 606	5,589 ± 1,271*
CDCA	15.2 ± 3.3	42 ± 14	17.4 ± 3.1	26 ± 6
DCA	148 ± 33	184 ± 87	66 ± 22	94 ± 32
7oxoDCA	188 ± 55	2,306 ± 1,488	985 ± 455	6,477 ± 1,679*
HDCA	25 ± 5	33 ± 12	33 ± 8	82 ± 23
αMCA	67 ± 17	425 ± 285	356 ± 91	2,685 ± 749*
βMCA	904 ± 145	276 ± 104*	2,693 ± 619	2,423 ± 511
ωMCA	888 ± 149	937 ± 479	1,268 ± 356	3,178 ± 763
UDCA	75 ± 10	77 ± 37	103 ± 26	249 ± 52*
7oxoLCA	6.8 ± 1.3	76 ± 38	28 ± 5	63 ± 17
αMCA/βMCA	0.18 ± 0.10	0.60 ± 0.09*	0.13 ± 0.02	0.96 ± 0.13*
UDCA/7oxoLCA	32 ± 12	4.5 ± 2.4*	5.0 ± 1.1	6.0 ± 1.0
CDCA/7oxoLCA	3.9 ± 1.6	1.86 ± 0.97*	0.91 ± 0.12	0.74 ± 0.12
C‐Tau	2,993 ± 1,687	2,091 ± 1,778	59,664 ± 15,995	66,470 ± 11,753
CDC‐Tau	76 ± 48	78 ± 53	2,355 ± 515	2,667 ± 536
DC‐Tau	200 ± 106	289 ± 149	3,843 ± 1,822	2,367 ± 735
LC‐Tau	16.0 ± 0.7	1.81 ± 0.70	50 ± 13	40 ± 9
αMCA‐Tau + βMCA‐Tau	1,854 ± 851	2,878 ± 1,923	30,702 ± 6,384	29,952 ± 6,056
ωMCA‐Tau	2,133 ± 1,324	1,286 ± 1,088	32,951 ± 6,074	28,000 ± 4,968
UDC‐Tau	103 ± 60	16.1 ± 9.8*	2,754 ± 531	886 ± 489*
7oxoLC‐Tau	9.1 ± 3.3	187 ± 110	560 ± 123	3,101 ± 935*
UDC‐Tau/7oxoLC‐Tau	158 ± 149	0.72 ± 0.22*	6.5 ± 1.1	0.40 ± 0.22*
CDC‐Tau/7oxoLC‐Tau	122 ± 120	0.78 ± 0.12*	5.6 ± 1.3	1.09 ± 0.11*
Sum primary BA	1,588 ± 179	3,962 ± 2,156	5,174 ± 1,266	10,971 ± 2,449
Sum primary BA‐Tau	5,026 ± 2,272	5,063 ± 3,625	95,475 ± 19,081	99,975 ± 17,536

*Note*: The results represent mean ± SEM (nmol·L^−1^ and fmol·mg^−1^ for plasma and liver, respectively). Analyte concentrations defined by a S/N ≤ 3 represent the LLOD of the UHPLC–MS/MS method. Samples yielding a concentration below LLOD were included as LLOD/2 (nmol·L^−1^ and fmol·mg^−1^, respectively) in the calculations of a specific analyte. Blue‐ and yellow‐coloured boxes indicate statistically significant increases and decreases, respectively. Unequal group sizes reflect exclusion of one 11LKO animal due to unexpected health issues prior to reaching the age for the experiment.

Abbreviations: 11LKO, liver‐specific *Hsd11b1* knockout; CTRL, control littermates.

* *P* < 0.05, significantly different as indicated; non‐parametric, Mann–Whitney *U* test (two‐tailed).

### 3.4 *H6pd*KO mice exhibit moderately decreased 11β‐HSD1 oxoreduction activity

**FIGURE 4:**
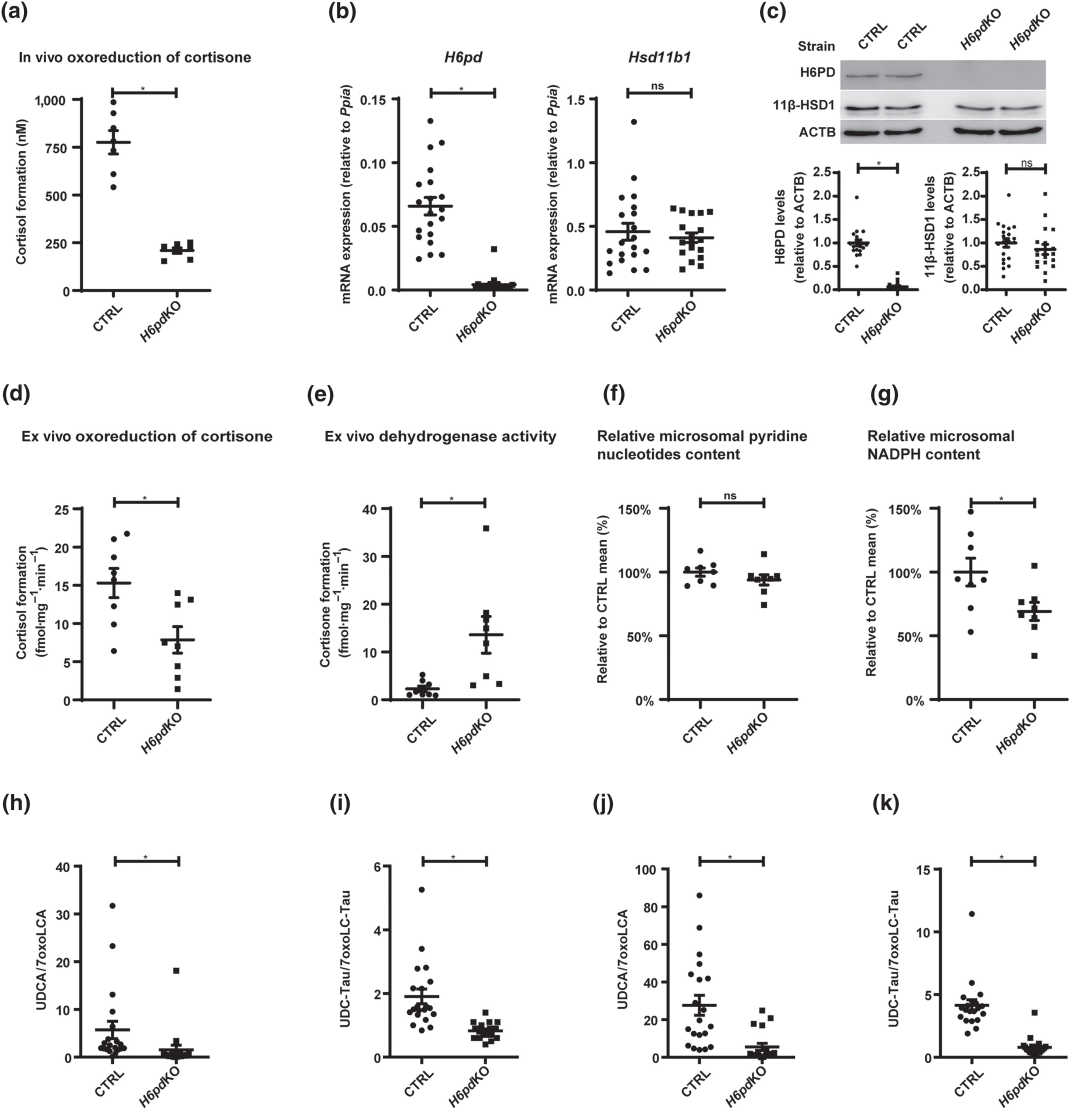
Characterization of 11β‐HSD1 expression and activity and plasma and liver tissue UDCA/7oxoLCA and UDC‐Tau/7oxoLC‐Tau ratios in *H6pd*KO mice. (a) Estimation of the cortisone to cortisol conversion *in vivo*, determined as plasma concentration (nmol·L^−1^) of cortisol 10 min after i.p. administration of 5 mg·kg^−1^ of cortisone (in DMSO) (CTRL *n* = 7; *H6pd*KO *n* = 7). (b) mRNA and (c) Western blot and semi‐quantitative analysis of protein levels of H6PD and 11β‐HSD1 in CTRL and *H6pd*KO animals (CTRL *n* = 20; *H6pd*KO *n* = 18). (d) Conversion of cortisone to cortisol and (e) of cortisol to cortisone determined ex vivo in mouse liver tissue (CTRL *n* = 8; *H6pd*KO *n* = 8). (f) Reduced pyridine nucleotides content and (g) NADPH content in mouse liver microsomes (relative to the mean of the CTRL; CTRL *n* = 8; *H6pd*KO *n* = 8). (h) UDCA/7oxoLCA ratios and (i) UDC‐Tau/7oxoLC‐Tau ratios in plasma of *H6pd*KO mice (CTRL *n* = 20; *H6pd*KO *n* = 18); (j) UDCA/7oxoLCA ratios and (k) UDC‐Tau/7oxoLC‐Tau ratios in liver tissue of *H6pd*KO mice (CTRL *n* = 20; *H6pd*KO *n* = 18). Analyte concentrations defined by a S/N ≤ 3 represent the LLOD of the UHPLC–MS/MS method. Samples yielding a concentration below LLOD were included as LLOD/2 (nmol·L^−1^ and fmol·mg^−1^ for plasma and liver, respectively) in the calculations of a specific analyte. Results represent mean ± SEM. *P < 0.05 significantly different as indicated; non‐parametric, Mann–Whitney *U* test (two‐tailed). Unequal group sizes reflect exclusion of two *H6pd*KO animals from further analysis due to the occurrence of liver cysts

Previous characterization of *H6pd*KO mice suggested, based on experiments using microsomal preparations, a complete loss of 11β‐HSD1 oxoreduction activity (Lavery et al., 2006). To assess the conversion of 11‐oxo‐ to 11β‐hydroxyglucocorticoids *in vivo*, *H6pd*KO mice and control littermates received cortisone i.p. and were killed 10 min later, followed by measuring formed cortisol. 11β‐HSD1 oxoreduction activity was reduced to approximately 60% of the level in control mice (Figure 4a), despite comparable hepatic 11β‐HSD1 mRNA and protein expression (Figure 4b,c). As expected, H6PD mRNA and protein expression were abolished in *H6pd*KO. Similarly, *ex vivo* oxoreduction was reduced in liver of *H6pd*KO mice to half the activity of controls (Figure 4d). There was a corresponding increase in dehydrogenase activity to approximately fivefold the level in control liver (Figure 4e). The ratio of oxoreduction to dehydrogenase activity was estimated to be about five in CTRL and 0.5 in *H6pd*KO liver tissue. Similar experiments in white adipose tissue showed approximately 40% residual oxoreduction activity in *H6pd*KO compared to CTRL, whilst dehydrogenase activity increased 30‐fold to 40‐fold (Figure S5). Thus, in contrast to the expectation of a complete loss of 11β‐HSD1 oxoreduction activity, these results revealed a moderately decreased oxoreduction in *H6pd*KO mice despite an increase in dehydrogenase activity.

An estimation of the impact of the lack of H6PD on NADPH levels in the ER using liver microsomes indicated that the content of total reduced pyridine nucleotides (NADPH + NADH) did not differ between control and *H6pd*KO (Figure 4f), but NADPH content of *H6pd*KO mouse liver microsomes was moderately lower by approximately 30% compared to control (Figure 4g).

### 3.5 The UDC‐Tau/7oxoLC‐Tau ratio detects decreased 11β‐HSD1 oxoreductase activity in *H6pd*KO mice

Next, plasma and liver tissue bile acid profiles between *H6pd*KO and control mice were compared. Unlike in 11KO, primary and taurine‐conjugated bile acids were not generally elevated in plasma of *H6pd*KO mice (Table 3). 7oxoLCA was 1.8‐fold higher in *H6pd*KO plasma, whilst UDCA was threefold lower and CDCA not different between the two genotypes. The ratio of CDCA/7oxoLCA only tended lower, whereas UDCA/7oxoLCA was 3.7‐fold lower in *H6pd*KO plasma (Figure 4h). 7oxoLC‐Tau was about ninefold higher in *H6pd*KO plasma than in CTRL, whereas UDC‐Tau was not different and CDC‐Tau tended to increase. Both ratios CDC‐Tau/7oxoLC‐Tau and UDC‐Tau/7oxoLC‐Tau (Figure 4h; see also Figure S6) were significantly lower in *H6pd*KO plasma. In agreement with 11KO and 11LKO, these observations were supported by a similar bile acid profile in liver tissue with almost threefold increased 7oxoLCA and twofold decreased UDCA in *H6pd*KO but no difference in CDCA (Table 3, Figure S7). The CDCA/7oxoLCA and UDCA/7oxoLCA ratios were twofold and fivefold lower, respectively, in *H6pd*KO liver tissue (Table 3, Figure 4i). The levels of the taurine‐conjugated bile acids were about an order of magnitude higher than those of their free forms. 7oxoLC‐Tau was fourfold higher in *H6pd*KO compared to control, but due to inter‐individual variation, the value did not reach significance. CDC‐Tau was not different between the two genotypes, whilst UDC‐Tau decreased twofold. The ratios CDC‐Tau/7oxoLC‐Tau and UDC‐Tau/7oxoLC‐Tau (Figure 4i; see also Figure S7) were threefold and fivefold lower, respectively, in *H6pd*KO liver tissue.

**TABLE 3:** Bile acid profiles in plasma and liver of *H6pd*KO mice

Analyte	Plasma (nmol·L^−1^)		Liver (fmol·mg^−1^)	
	CTRL *H6pd*KO	*H6pd*KO	CTRL	*H6pd*KO
	(*n* = 20)	(*n* = 18)	(*n* = 20)	(*n* = 18)
CA	385 ± 245	214 ± 100	6,174 ± 1,321	4,324 ± 1,460
CDCA	8.8 ± 4.0	8.6 ± 1.5	304 ± 54	280 ± 55
DCA	187 ± 39	113 ± 13*	244 ± 31	166 ± 23
7oxoDCA	142 ± 97	167 ± 88	4,332 ± 1,175	4,555 ± 1,647
HDCA	18.4 ± 5.7	17.5 ± 2.5	350 ± 67	277 ± 39
αMCA	32 ± 16	37 ± 18	2,921 ± 688	2,544 ± 596
βMCA	259 ± 138	54 ± 19*	9,297 ± 1,932	2,652 ± 512*
ωMCA	71 ± 26	43 ± 9	2,154 ± 367	1,087 ± 183
UDCA	45 ± 24	15.4 ± 5.7*	365 ± 57	174 ± 48*
7oxoLCA	12.0 ± 2.4	20 ± 2*	37 ± 13	103 ± 28
αMCA/βMCA	0.12 ± 0.01	0.51 ± 0.06*	0.23 ± 0.02	0.98 ± 0.15*
UDCA/7oxoLCA	5.7 ± 1.8	1.53 ± 0.99*	28 ± 5	5.5 ± 1.9*
CDCA/7oxoLCA	1.38 ± 0.53	0.77 ± 0.39	20 ± 3	10.5 ± 3.5*
C‐Tau	5,602 ± 3,712	9,620 ± 8,041	565,130 ± 129,003	394,188 ± 108,185
CDC‐Tau	234 ± 133	429 ± 317	28,597 ± 7,271	30,359 ± 7,770
DC‐Tau	381 ± 238	565 ± 404	25,629 ± 5,137	19,979 ± 5,696
LC‐Tau	4.7 ± 3.9	12.2 ± 10.7	947 ± 67	997 ± 114
αMCA‐Tau + βMCA‐Tau	1,247 ± 877	854 ± 733	354,892 ± 80,362	193,231 ± 45,864
ωMCA‐Tau	2,436 ± 1,627	2,974 ± 2,355	151,216 ± 38,734	80,001 ± 14,690
UDC‐Tau	228 ± 161	293 ± 244	22,609 ± 5,964	9,885 ± 2,777*
7oxoLC‐Tau	72 ± 46	651 ± 569	5,541 ± 1,045	22,711 ± 6,325
UDC‐Tau/7oxoLC‐Tau	1.91 ± 0.23	0.83 ± 0.06*	4.1 ± 0.4	0.80 ± 0.18*
CDC‐Tau/7oxoLC‐Tau	3.03 ± 0.32	1.70 ± 0.10*	5.0 ± 0.5	1.75 ± 0.24*
Sum primary BA	730 ± 426	333 ± 142	19,060 ± 3,852	9,975 ± 2,444
Sum primary BA‐Tau	14,127 ± 9,474	22,922 ± 19,167	971,227 ± 220,934	627,664 ± 162,026

*Note*: The results represent mean ± SEM (nmol·L^−1^ and fmol·mg^−1^ for plasma and liver, respectively). Analyte concentrations defined by a S/N ≤ 3 represent the LLOD of the UHPLC–MS/MS method. Samples yielding a concentration below LLOD were included as LLOD/2 (nmol·L^−1^ and fmol·mg^−1^, respectively) in the calculations of a specific analyte. Blue‐ and yellow‐coloured boxes indicate statistically significant increases and decreases, respectively. Unequal group sizes reflect exclusion of two *H6pd*KO animals from further analysis due to the occurrence of liver cysts.

Abbreviations: CTRL, control littermates; *H6pd*KO, global *H6pd* knockout.

* *P* < 0.05, significantly different as indicated; non‐parametric, Mann–Whitney *U* test (two‐tailed).

As seen in 11KO and 11LKO, the ratio of αMCA/βMCA was significantly higher in *H6pd*KO plasma (fourfold) and liver tissue (fivefold). In *H6pd*KO mice, this was due to significantly lower βMCA. αMCA was not different between the genotypes (Table 3).

### 3.6 The UDC‐Tau/7oxoLC‐Tau ratio detects pharmacologically diminished 11β‐HSD1 activity

The UDC‐Tau/7oxoLC‐Tau ratio detected the reduced 11β‐HSD1 activity due to genetic alteration of its expression (11KO, 11LKO) or reaction direction (*H6pd*KO). To see if enzyme inhibition without genetic manipulation also can be detected, the known 11β‐HSD1 inhibitor carbenoxolone was used. Inhibition by carbenoxolone of 11β‐HSD1‐dependent metabolism of 7oxoLCA was first assessed *in vitro*. HEK‐293 cells stably expressing murine 11β‐HSD1 (MO1F cells) showed a time‐dependent conversion of 7oxoLCA to comparable amounts of UDCA and CDCA, with almost complete metabolism of 7oxoLCA after 24 h (Figure S8). Incubation with carbenoxolone almost fully blocked the formation of UDCA and CDCA, supporting the use of product/substrate ratios as markers of 11β‐HSD1 activity.

**FIGURE 5:**
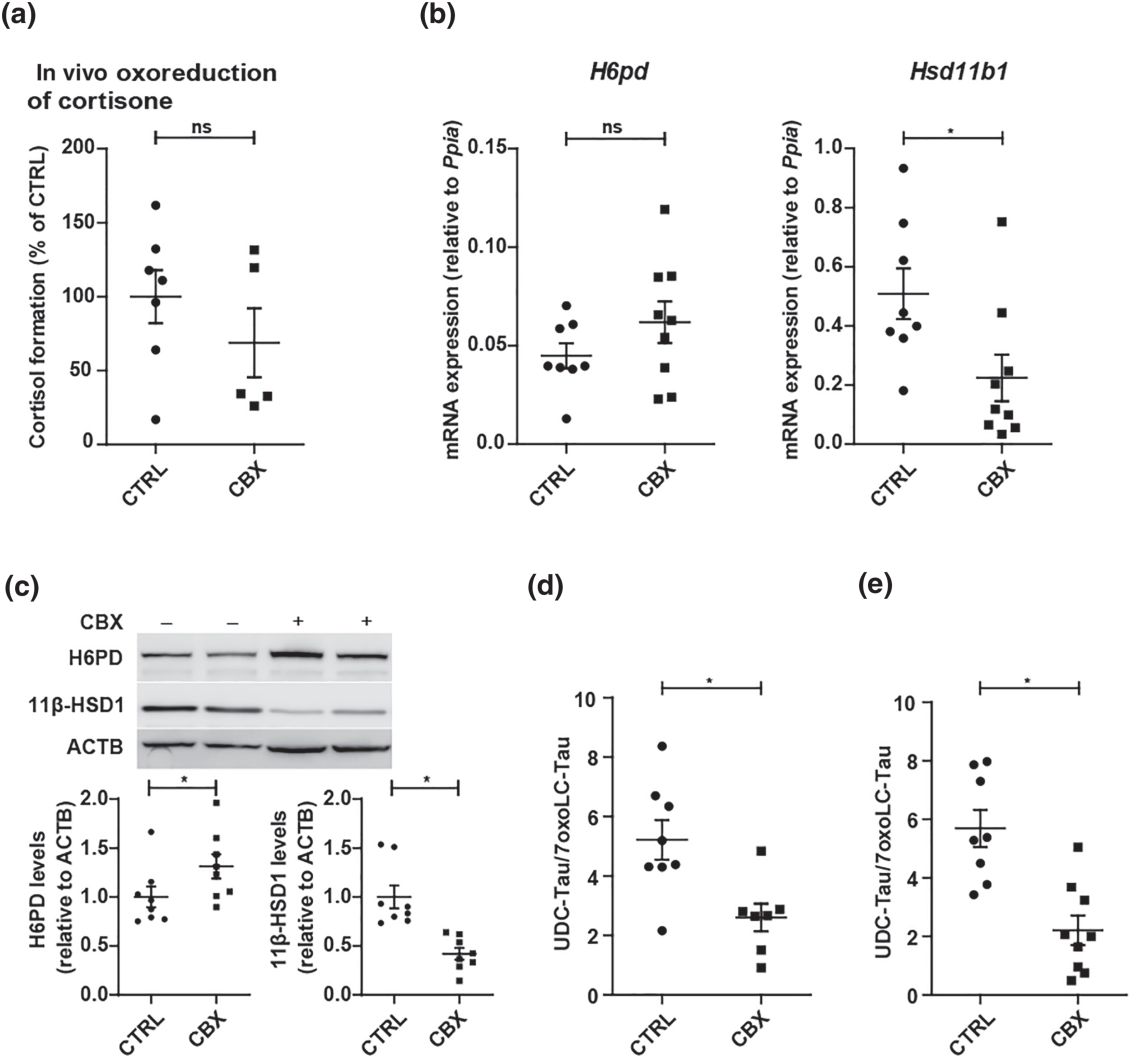
Characterization of 11β‐HSD1 expression and activity and plasma and liver tissue UDC‐Tau/7oxoLC‐Tau ratios in carbenoxolone‐treated mice. Control mice were treated with the pharmacologic 11β‐HSD1 inhibitor carbenoxolone (CBX, 100 mg·kg^−1^·day^−1^, i.p.) or PBS (CTRL). (a) Conversion of cortisone to cortisol *in vivo*, measured after i.p. administration of 5 mg·kg^−1^ of cortisone (in DMSO) (CTRL *n* = 7; CBX *n* = 5). (b) mRNA expression of *H6pd* and *Hsd11b1* in CTRL‐ and CBX‐treated animals (CTRL *n* = 8; CBX *n* = 9). (c) Western blot and semi‐quantitative analysis of protein levels of H6PD and 11β‐HSD1 in CTRL‐ and CBX‐treated mice (CTRL *n* = 8; CBX *n* = 8). (d) UDC‐Tau/7oxoLC‐Tau ratios in CTRL mice treated with PBS (CTRL) or the pharmacologic 11β‐HSD1 inhibitor carbenoxolone (CBX 100 mg·kg^−1^·day^−1^, i.p.) (CTRL *n* = 8; CBX *n* = 9) in plasma and (e) in liver tissue. Analyte concentrations defined by a S/N ≤ 3 represent the LLOD of the UHPLC–MS/MS method. Samples yielding a concentration below LLOD were included as LLOD/2 (nmol·L^−1^ and fmol·mg^−1^ for plasma and liver, respectively) in the calculations of a specific analyte. Results represent mean ± SEM. No outliers were excluded. *P < 0.05 significantly different as indicated; non‐parametric, Mann–Whitney *U* test (two‐tailed). Unequal group sizes reflect exclusion of one animal of the CTRL group due to unexpected health issues prior to the experiment and exclusion of two plasma samples of the CBX group due to insufficient collection of blood sample volume

Next, mice were treated i.p. with 100 mg·kg^−1^·day^−1^ of carbenoxolone for 4 days. carbenoxolone treatment decreased cortisone to cortisol conversion by approximately 30%, revealing moderate 11β‐HSD1 inhibition (Figure 5a). Interestingly, analysis of mRNA showed a reduction in *Hsd11b1* mRNA expression and a trend for increased *H6pd* mRNA (Figure 5b). This was corroborated by similar effects on H6PD (30% increase) and 11β‐HSD1 (50–60% decrease) protein expression (Figure 5c), suggesting that besides pharmacological inhibition, a reduced expression contributed to the lower 11β‐HSD1 activity in this model.

Analysis of bile acid profiles revealed that the sum of free primary bile acids tended to be lower (2.5‐fold) in plasma upon carbenoxolone treatment, whereas taurine‐conjugated primary bile acids seemed to be not affected (Table 4). 7oxoLCA concentrations were below the lower limit of detection (LLOD) in plasma and liver tissue samples in this mouse cohort, so the respective product/substrate ratios with UDCA and CDCA could not be calculated. In plasma of carbenoxolone‐treated mice, 7oxoLC‐Tau tended to increase, whilst UDC‐Tau and CDC‐Tau were not affected by carbenoxolone (Figure S9). Nevertheless, CDC‐Tau/7oxoLC‐Tau and UDC‐Tau/7oxoLC‐Tau (Figure 5d; see also Figure S9) were 1.6‐fold and twofold lower in plasma of carbenoxolone‐treated mice. In liver tissue, only UDC‐Tau/7oxoLC‐Tau was predictive for decreased 11β‐HSD1 activity (2.6‐fold decreased; Table 4, Figures 5e and S10).

**TABLE 4:** Bile acid profiles in plasma and liver in mice with decreased 11β‐HSD1 oxoreduction activity by pharmacologic inhibition using carbenoxolone

Analyte	Plasma (nmol·L^−1^)		Liver (fmol·mg^−1^)	
	CTRL	CBX	CTRL	CBX
	(*n* = 8)	(*n* = 7)	(*n* = 8)	(*n* = 9)
CA	1,000 ± 795	408 ± 275	513 ± 84	329 ± 71
CDCA	145 ± 120	11.0 ± 2.3	242 ± 17	114 ± 45
DCA	592 ± 245	169 ± 62*	186 ± 15	61 ± 21*
7oxoDCA	237 ± 178	281 ± 212	46 ± 17	88 ± 50
HDCA	146 ± 90	26 ± 12	224 ± 17	113 ± 34*
αMCA	162 ± 110	42 ± 17	288 ± 33	129 ± 42*
βMCA	659 ± 481	318 ± 179	1,608 ± 136	4,409 ± 1,557
ωMCA	238 ± 134	47 ± 25	306 ± 25	580 ± 183
UDCA	226 ± 126	39 ± 18*	99 ± 14	17.8 ± 6.2*
7oxoLCA	NA	NA	NA	NA
αMCA/βMCA	0.24 ± 0.05	0.28 ± 0.15	0.18 ± 0.02	0.06 ± 0.02*
UDCA/7oxoLCA	NA	NA	NA	NA
CDCA/7oxoLCA	NA	NA	NA	NA
C‐Tau	7,002 ± 4,900	9,530 ± 6,466	63,049 ± 11,354	48,334 ± 9,673
CDC‐Tau	339 ± 223	265 ± 175	5,274 ± 533	3,975 ± 857
DC‐Tau	348 ± 275	222 ± 124	6,723 ± 719	2,114 ± 766*
LC‐Tau	8.8 ± 5.6	2.01 ± 1.22	1,271 ± 127	541 ± 81*
αMCA‐Tau + βMCA‐Tau	1,876 ± 1,853	1,383 ± 943	55,442 ± 6,370	63,438 ± 12,465
ωMCA‐Tau	3,124 ± 1,998	12,920 ± 11,548	25,304 ± 3,379	32,334 ± 8,484
UDC‐Tau	296 ± 221	332 ± 251	4,071 ± 501	1,125 ± 174*
7oxoLC‐Tau	84 ± 56	125 ± 94	836 ± 178	760 ± 209
UDC‐Tau/7oxoLC‐Tau	5.2 ± 0.7	2.6 ± 0.5*	5.7 ± 0.6	2.2 ± 0.5*
CDC‐Tau/7oxoLC‐Tau	2.6 ± 0.4	1.60 ± 0.38*	7.7 ± 1.0	6.2 ± 1.0
Sum primary BA	2,192 ± 1,630	818 ± 483	2,463 ± 192	4,869 ± 1,611
Sum primary BA‐Tau	9,513 ± 7,033	11,511 ± 7,034	127,836 ± 17,002	116,873 ± 15,450

*Note*: The results represent mean ± SEM (nmol·L^−1^ and fmol·mg^−1^ for plasma and liver, respectively). Analyte concentrations defined by a S/N ≤ 3 represent the LLOD of the UHPLC–MS/MS method. Samples yielding a concentration below LLOD were included as LLOD/2 (nmol·L^−1^ and fmol·mg^−1^, respectively) in the calculations of a specific analyte. Yellow‐coloured boxes indicate statistically significant decreases. Unequal group sizes reflect exclusion of one animal of the CTRL group due to unexpected health issues prior to the experiment and exclusion of two plasma samples of the carbenoxolone (CBX group due to insufficient collection of blood sample volume.

Abbreviations: CBX, mice treated with the pharmacologic 11β‐HSD1 inhibitor carbenoxolone (100 mg·kg^−1^·day^−1^, i.p.); CTRL, control mice treated with PBS; NA, not analysed, if most values were below LLOD.

* *P* < 0.05, significantly different as indicated; non‐parametric, Mann–Whitney *U* test (two‐tailed).

In contrast to the other three mouse models, the αMCA/βMCA ratio was unchanged in plasma and even threefold lower in liver tissue; thus, this ratio is not indicative of altered 11β‐HSD1 activity.

## 4 DISCUSSION

This proof‐of‐concept study proposes that the UDC‐Tau/7oxoLC‐Tau ratio can serve as a biomarker for decreased 11β‐HSD1 activity *in vivo*. The UDC‐Tau/7oxoLC‐Tau ratio in plasma and liver tissue successfully detected complete loss of 11β‐HSD1 activity in 11KO mice, loss of hepatic 11β‐HSD1 activity in 11LKO mice and moderately decreased oxoreduction activity in *H6pd*KO‐ and carbenoxolone‐treated mice. Of note, the four models differed with respect to their genetic background (11KO, 11LKO and *H6pd*KO on a mixed C57BL/6J/129SvJ background, carbenoxolone group were C57BL/6JRj) that can affect lipid and bile acid homeostasis (Jolley et al., 1999), feeding regimen (11KO and 11LKO fasted overnight, the other two models *ad libitum* feeding; some differences in the composition of the chow) and environment (different animal facilities) that can impact the microbiome and thereby influence bile acid homeostasis (Rausch et al., 2016). A biomarker reporting decreased 11β‐HSD1 oxoreduction activity in plasma opens the possibility for non‐invasive applications in preclinical studies of pharmacological inhibitors for potential therapeutic applications; whether it can also be used to explore the pathophysiological role of 11β‐HSD1 in situations of elevated activity remains to be investigated (Gathercole et al., 2013). Determination of this ratio in liver tissue at the end of the study can provide additional information.

The plasma UDCA/7oxoLCA ratio was also a marker for decreased 11β‐HSD1 activity. However, because the levels of free bile acids are lower than those of their taurine‐conjugated forms and were below the LLOD in some mice, this is likely to be less useful practically than the ratio of the taurine‐conjugated metabolites. Whilst in mice and rats taurine‐conjugated bile acids are predominant and the UDC‐Tau/7oxoLC‐Tau ratio is easier to assess, the ratio of the free UDCA/7oxoLCA has the advantage to be species independent, as glycine‐conjugated bile acids are predominant in human and other higher mammals (Alnouti et al., 2008; Garcia‐Canaveras et al., 2012; Penno et al., 2014). Improvements of the analytical sensitivity may be achieved by measuring just UDCA and 7oxoLCA, using larger sample volumes, and optimizing extraction specifically for these two bile acids, which should permit this ratio to be a good and reliable species‐independent marker.

Although murine 11β‐HSD1 converts 7oxoLC‐Tau to UDC‐Tau and CDC‐Tau (Odermatt et al., 2011), the UDC‐Tau/7oxoLC‐Tau ratio was superior to the CDC‐Tau/7oxoLC‐Tau for detecting decreased 11β‐HSD1 activity. A possible explanation may be the significant contribution of de novo CDCA synthesis to the circulating and liver tissue levels of CDC‐Tau, whereas UDC‐Tau and 7oxoLC‐Tau are primarily formed from gut microbiota‐derived UDCA and 7oxoLCA.

Interestingly, an increase in the ratio of αMCA/βMCA, formed by cytochrome P450 2C70 from CDCA and UDCA, respectively (Takahashi et al., 2016), nicely detected the decreased 11β‐HSD1 activity in the three genetically modified mouse models. However, in carbenoxolone‐treated mice, this ratio was not changed in plasma and showed an opposite change in liver. Plausibly, carbenoxolone inhibits cytochrome P450 2C70 or decreases its expression. Carbenoxolone might also affect gut microbiota as it was earlier shown to alter colonic mucus (Finnie et al., 1996). This merits future investigation.

Pharmacological treatment using carbenoxolone led to approximately 30% decreased 11β‐HSD1 activity. It needs to be noted that the level of 11β‐HSD1 inhibition is an estimation and it was determined at one given time point (i.e., at about 8 am) and the formation of cortisol upon injection of cortisone was determined after 10 min. Nevertheless, inhibition of 11β‐HSD1 could be demonstrated and the bile acid biomarker detected the decreased activity. The results suggested that besides direct inhibition, a reduced enzyme expression contributed to the decreased activity. carbenoxolone also inhibits 11β‐HSD2 (Stewart et al., 1990). However, our preliminary observations suggest that this enzyme does not accept CDCA and UDCA as substrates, therefore unlikely affecting the bile acid ratios of interest.

*H6pd*KO mice retained approximately 50% of 11β‐HSD1 oxoreduction activity measured in control animals. This is consistent with observations made in isolated macrophage from *H6pd*KO mice, which also retained about 50–60% 11β‐HSD1 oxoreduction activity (Marbet et al., 2018). Based on earlier experiments using liver microsomes (Lavery et al., 2006), it was anticipated that in the absence of H6PD, 11β‐HSD1 would function exclusively as dehydrogenase and the effect on the respective bile acid ratios would be comparable with that in 11KO mice, yet the accumulation of 7oxo metabolites and the ratios derived from them clearly were less pronounced. These findings indicate the existence of a yet unknown mechanism generating NADPH in the ER capable of driving 11β‐HSD1 reaction direction towards oxoreduction activity. This is supported by the continued presence of NADPH in the *H6pd*KO liver, albeit at reduced levels, seen previously (Rogoff et al., 2010) and also found here. A possible candidate for generating NADPH within the ER includes luminal 6‐phosphogluconate dehydrogenase (Bublitz et al., 1987). However, the gene encoding this enzyme still remains to be identified.

The mild cholestasis phenotype of 11KO mice with 10‐fold to 20‐fold increased plasma bile acids (Table 1, Penno et al., 2014) raises some concerns that pharmacological 11β‐HSD1 inhibition might induce cholestasis. In paediatric patients with adrenal insufficiency and cholestasis, glucocorticoid treatment reversed the hepatic phenotype (Al‐Hussaini et al., 2012; Cheung et al., 2003), indicating a direct role of glucocorticoids in maintaining bile acid homeostasis. However, no evidence for cholestasis was seen in the present study when 11β‐HSD1 was inhibited by carbenoxolone and there was only a trend increase of total free but not conjugated bile acids in 11LKO mice and no change of total bile acids in *H6pd*KO mice. These observations do not support concerns of a general risk of cholestasis upon inhibition of 11β‐HSD1. The more pronounced effect on plasma and liver tissue bile acid profiles in 11KO mice may be explained by the fact that they lack 11β‐HSD1 during all stages of life and throughout the enterohepatic circuit and also by altered hypothalamus–pituitary–adrenal axis, whereas 11LKO only lack hepatic 11β‐HSD1, and *H6pd*KO‐ and carbenoxolone‐treated mice retain partial 11β‐HSD1 activity.

A suitable biomarker reporting the *in vivo* 11β‐HSD1 activity in health and disease situations or upon pharmacological interventions could greatly facilitate such studies. The currently used urinary (tetrahydrocorticosterone + allo‐tetrahydrocorticosterone)/tetrahydro‐11‐dehydrocorticosterone ratio has limited value as it can lead to erroneous conclusions because of interference through altered 11β‐HSD2 and 5α‐reductase activities and feedback modulation. Furthermore, it needs 24‐h urine sampling that due to small collection volume and contamination by food and faeces and the stress of metabolic cage housing may lead to erroneous results.

Although our data support the UDC‐Tau/7oxoLC‐Tau ratio as a useful *in vivo* marker of 11β‐HSD1 activity, our study has several limitations:‐ (1) the present study included only male mice at 10–15 weeks of age and it will be important to study also mice at both young and very old age that may exhibit metabolic differences as well as female mice, being mindful of the effect of the oestrous cycle on bile acid homeostasis (Papacleovoulou et al., 2011); (2) the impact of feeding and diet should be studied; (3) samples were taken in the morning between 7 and 10 am and the influence of circadian rhythm and/or stress should be assessed; (4) in case of pharmacological inhibition, a possible interference of the compound with hepatic enzymes and transporters that also are involved in bile acid homeostasis needs to be kept in mind; (5) the impact of the microbiome on the production of UDCA and 7oxoLCA needs to be investigated; it has been shown that 11β‐HSD1 deficiency alters the microbiome in a diet‐specific manner (Johnson et al., 2017); (6) disease models with altered 11β‐HSD1 activity should be studied and (7) the sensitivity of the LC–MS/MS‐based quantification method can be increased by measuring specifically the bile acid metabolites needed for the ratio and by increasing sample volume and optimizing extraction. In follow‐on research, the predictivity of the UDC‐Tau/7oxoLC‐Tau ratio for detecting altered 11β‐HSD1 activity should be investigated in mouse models addressing the abovementioned factors. Finally, experiments in humans are required to establish whether such a bile acid ratio is a useful biomarker to detect altered 11β‐HSD1 activity upon pharmacological treatment or in disease situations.

## AUTHOR CONTRIBUTIONS

M.W., S.S., D.V.K., J.B. and P.K. designed and conducted the experiments, analysed data, and reviewed the paper; K.E.C. consulted for the study and reviewed the paper; G.G.L. consulted for the study and reviewed the paper; and A.O. designed the experiments, supervised the study and wrote the paper.

## CONFLICT OF INTEREST

The authors have nothing to disclose.

## DECLARATION OF TRANSPARENCY AND SCIENTIFIC RIGOUR

This Declaration acknowledges that this paper adheres to the principles for transparent reporting and scientific rigour of preclinical research as stated in the *BJP* guidelines for Design & Analysis, Immunoblotting and Immunochemistry and Animal Experimentation, and as recommended by funding agencies, publishers, and other organizations engaged with supporting research.

## Supporting information

**Table S1.** Effect sizes of bile acid profiles measured by UHPLC‐MS/MS in plasma and liver samples were determined for mice with global *Hsd11b1* knockout (11KO), liver‐specific *Hsd11b1* knockout (11LKO), global *H6pd* knockout (*H6pd*KO) compared to the respective control littermates. Additionally, mice treated with the pharmacological inhibitor carbenoxolone (CBX) were compared to untreated control mice. Results represent the effect size calculated based on Cohen's d effect size (ES d) with correction of unequal sample sizes for the analysis of data from non‐parametric analysis (Mann‐Whitney U‐test) calculated as Hedges g (ES g) and corresponding confidence intervals of the effect size (± CI). NA, not analysed.
**Figure S1.** Decreased 11β‐HSD1 bile acid product to substrate ratios in plasma of 11KO mice. 11β‐HSD1 bile acid substrates (7oxoLCA, 7oxoLC‐Tau) and products (UDCA, CDCA and their taurine conjugated forms) were measured in plasma of 11KO mice (nmol·L^−1^). Calculation of the product to substrate ratios attenuated the large animal‐to‐animal variations and detected the lack of 11β‐HSD1 oxoreduction activity. a) Plasma concentrations of CTRL (n = 18) and 11KO mice (n = 17) for 7oxoLCA, CDCA, UDCA and the corresponding ratios; and b) for 7oxoLCTau, CDC‐Tau, UDC‐Tau and the corresponding ratios. The results represent mean ± SEM. No outliers were excluded. Analyte concentrations defined by a S/N ≤ 3 represent the LLOD of the UHPLC‐MS/MS method. Samples yielding a concentration below LLOD were included as LLOD/2 in the calculations of a specific analyte. *P<0.05 significantly different as indicated; non‐parametric, Mann‐Whitney U‐test (two‐tailed). Unequal group sizes reflect exclusion of one plasma sample due to insufficient collection of blood sample volume.
**Figure S2.** Decreased 11β‐HSD1 bile acid product to substrate ratios in liver tissue of 11KO mice. 11β‐ HSD1 bile acid substrates (7oxoLCA, 7oxoLC‐Tau) and products (UDCA, CDCA and their taurine conjugated forms) were measured in liver tissue of 11KO mice (fmol·mg^−1^). Calculation of the product to substrate ratios attenuated the large animal‐to‐animal variations and detected the lack of 11β‐HSD1 oxoreduction activity. a) Liver tissue concentrations of CTRL (n = 9) and 11KO mice (n = 9) for 7oxoLCA, CDCA, UDCA and the corresponding ratios; and b) for 7oxoLC‐Tau, CDC‐Tau, UDC‐Tau and the corresponding ratios. The results represent mean ± SEM. No outliers were excluded. Analyte concentrations defined by a S/N ≤ 3 represent the LLOD of the UHPLC‐MS/MS method. Samples yielding a concentration below LLOD were included as LLOD/2 in the calculations of a specific analyte. *P<0.05 significantly different as indicated; non‐parametric, Mann‐Whitney U‐test (two‐tailed). Unequal group sizes reflect the availability of only nine livers due to the use of nine randomly assigned livers for gene expression analyses in a previous study.
**Figure S3.** Decreased 11β‐HSD1 bile acid product to substrate ratios in plasma of 11LKO mice. 11β‐HSD1 bile acid substrates (7oxoLCA, 7oxoLC‐Tau) and products (UDCA, CDCA and their taurine conjugated forms) were measured in plasma of 11LKO mice (nmol·L^−1^). Calculation of the product to substrate ratios attenuated the large animal‐to‐animal variations and detected the 4 decreased 11β‐HSD1 oxoreduction activity. a) Plasma concentrations for 7oxoLCA, CDCA, UDCA and the corresponding ratios, and b) for 7oxoLC‐Tau, CDC‐Tau, UDC‐Tau and the corresponding ratios of CTRL (n = 17) and 11LKO mice (n = 16). The results represent mean ± SEM. No outliers were excluded. Analyte concentrations defined by a S/N ≤ 3 represent the LLOD of the UHPLC‐MS/MS method. Samples yielding a concentration below LLOD were included as LLOD/2 in the calculations of a specific analyte. *P<0.05 significantly different as indicated; non‐parametric, Mann‐Whitney U‐test (two‐tailed). Unequal group sizes reflect exclusion of one 11LKO animal due to unexpected health issues prior to reaching the age for the experiment.
**Figure S4.** Decreased 11β‐HSD1 bile acid product to substrate ratios in liver tissue of 11LKO mice. 11β‐ HSD1 bile acid substrates (7oxoLCA, 7oxoLC‐Tau) and products (UDCA, CDCA and their taurine conjugated forms) were measured in liver tissue of 11LKO mice (fmol·mg^−1^). Calculation of the product to substrate ratios attenuated the large animal‐to‐animal variations and detected the decreased 11β‐HSD1 oxoreduction activity. a) Liver tissue concentrations for 7oxoLCA, CDCA, UDCA and the corresponding ratios, and b) for 7oxoLC‐Tau, CDC‐Tau, UDC‐Tau and the corresponding ratios of CTRL (n = 17) and 11LKO mice (n = 16). The results represent mean ± SEM. No outliers were excluded. Analyte concentrations defined by a S/N ≤ 3 represent the LLOD of the UHPLC‐MS/MS method. Samples yielding a concentration below LLOD were included as LLOD/2 in the calculations of a specific analyte. *P<0.05 significantly different as indicated; non‐parametric, Mann‐Whitney U‐test (two‐tailed). Unequal group sizes reflect exclusion of one 11LKO animal due to unexpected health issues prior to reaching the age for the experiment.
**Figure S5.** Decreased 11β‐HSD1 oxoreduction activity in white adipose tissue of *H6pd*KO mice. a) Estimation of the conversion of cortisone to cortisol, and b) of cortisol to cortisone measured *ex vivo* in mouse white adipose tissue (CTRL n = 7; *H6pd*KO n = 7). Results represent mean ± SEM, *P<0.05 significantly different as indicated; non‐parametric, Mann‐Whitney U‐test (twotailed). 5
**Figure S6.** Decreased 11β‐HSD1 bile acid product to substrate ratios in plasma of *H6pd*KO mice. 11β‐ HSD1 bile acid substrates (7oxoLCA, 7oxoLC‐Tau) and products (UDCA, CDCA and their taurine conjugated forms) were measured in plasma of *H6pd*KO mice. Calculation of the product to substrate ratios attenuated the large animal‐to‐animal variations and detected the decreased 11β‐HSD1 oxoreduction activity. a) Plasma concentrations (nmol·L^−1^) for 7oxoLCA, CDCA, UDCA and the corresponding ratios, and b) for 7oxoLC‐Tau, CDC‐Tau, UDC‐Tau and the corresponding ratios of CTRL (n = 20) and *H6pd*KO mice (n = 18). The results represent mean ± SEM. No outliers were excluded. Analyte concentrations defined by a S/N ≤ 3 represent the LLOD of the UHPLC‐MS/MS method. Samples yielding a concentration below LLOD were included as LLOD/2 in the calculations of a specific analyte. *P<0.05 significantly different as indicated; non‐parametric, Mann‐Whitney U‐test (two‐tailed). Unequal group sizes reflect exclusion of two *H6pd*KO animals from further analysis due to the occurrence of liver cysts.
**Figure S7.** Decreased 11β‐HSD1 bile acid product to substrate ratios in liver tissue of *H6pd*KO mice. 11β‐ HSD1 bile acid substrates (7oxoLCA, 7oxoLC‐Tau) and products (UDCA, CDCA and their taurine conjugated forms) were measured in liver tissue of *H6pd*KO mice. Calculation of the product to substrate ratios attenuated the large animal‐to‐animal variations and detected the decreased 11β‐HSD1 oxoreduction activity. a) Liver tissue concentrations (fmol·mg^−1^) for 7oxoLCA, CDCA, UDCA and the corresponding ratios, and b) for 7oxoLC‐Tau, CDC‐Tau, UDC‐Tau and the corresponding ratios of CTRL (n = 20) and *H6pd*KO mice (n = 18). The results represent mean ± SEM. No outliers were excluded. Analyte concentrations defined by a S/N ≤ 3 represent the LLOD of the UHPLC‐MS/MS method. Samples yielding a concentration below LLOD were included as LLOD/2 in the calculations of a specific analyte. *P<0.05 significantly different as indicated; non‐parametric, Mann‐Whitney U‐test (two‐tailed). Unequal group sizes reflect exclusion of two *H6pd*KO animals from further analysis due to the occurrence of liver cysts.
**Figure S8.** Metabolism of 7oxoLCA to UDCA and CDCA by murine 11β‐HSD1 and inhibition by carbenoxolone (CBX). HEK‐293 cells expressing murine 11β‐HSD1 (MO1F) were incubated with 400 nmol L^−1^ 7oxoLCA in the absence and presence of 5 μmol L^−1^ of CBX, followed by quantification of 6 substrate and products. a) 7oxoLCA concentration [nmol L^−1^] after 4 h and 24 h of incubation. b) CDCA formation represented as concentration [nmol L^−1^] after 4 h and 24h of incubation. c) UDCA formation represented as concentration [nmol L^−1^] after 4 h and 24 h of incubation. d) Representative western blot analysis of protein levels of 11β‐HSD1 in untransfected HEK‐293 cells and in MO1F cells. ACTB served as loading control. The results represent mean ± SD, n=5. *P<0.05 significantly different as indicated; non‐parametric, Mann‐Whitney U‐test (twotailed).
**Figure S9.** Decreased 11β‐HSD1 bile acid product to substrate ratios in plasma of mice treated with the inhibitor carbenoxolone (CBX). 11β‐HSD1 bile acid substrates (7oxoLCA, 7oxoLC‐Tau) and products (UDCA, CDCA and their taurine conjugated forms) were measured in plasma of control mice either treated with the pharmacologic 11β‐HSD1 inhibitor CBX (100 mg kg^−1^ d^−1^, *i.p.*) or PBS (CTRL) (CTRL n = 8; CBX n = 7). Calculation of the product/substrate ratios attenuated the large animal‐to‐animal variations and detected the diminished 11β‐HSD1 oxoreduction activity. a) Plasma concentrations (nmol·L^−1^) of 7oxoLCA, CDCA, UDCA and the corresponding ratios. Some values were below LLOD. b) Plasma concentrations (nmol·L^−1^) of 7oxoLC‐Tau, CDC‐Tau, UDC‐Tau and the corresponding ratios. The results represent mean ± SEM. No outliers were excluded. Analyte concentrations defined by a S/N of ≤ 3 represent the LLOD of the UHPLC‐MS/MS method. Samples yielding a concentration below LLOD were included as LLOD/2 in the calculations of a specific analyte. *P<0.05 significantly different as indicated; non‐parametric, Mann‐Whitney U‐test (two‐tailed). Unequal group sizes reflect exclusion of one animal of the CTRL group due to unexpected health issues prior to the experiment and exclusion of two plasma samples of the CBX group due to insufficient collection of blood sample volume.
**Figure S10.** Decreased 11β‐HSD1 bile acid product to substrate ratios in liver tissue of mice treated with the inhibitor carbenoxolone (CBX). 11β‐HSD1 bile acid substrates (7oxoLCA, 7oxoLC‐Tau) and products (UDCA, CDCA and their taurine conjugated forms) were measured in liver tissue of control mice either treated with the pharmacologic 11β‐HSD1 CBX (100 mg kg^−1^ d^−1^, *i.p.*) or PBS (CTRL) (CTRL n = 8; WT CBX n = 9). Calculation of the product/substrate ratios attenuated the large animal‐to‐animal variations and detected the diminished 11β‐HSD1 oxoreduction activity. a) Liver tissue concentrations (fmol·mg^−1^) of 7oxoLCA, CDCA, UDCA 7 and the corresponding ratios. Some values were below LLOD. b) Liver tissue concentrations (fmol·mg^−1^) of 7oxoLC‐Tau, CDC‐Tau, UDC‐Tau and the corresponding ratios. The results represent mean ± SEM. No outliers were excluded. Analyte concentrations defined by a S/N ≤ 3 represent the LLOD of the UHPLC‐MS/MS method. Samples yielding a concentration below LLOD were included as LLOD/2 in the calculations of a specific analyte. *P<0.05 significantly different as indicated; non‐parametric, Mann‐Whitney U‐test (two‐tailed). Unequal group sizes reflect exclusion of one animal of the CTRL group due to unexpected health issues prior to the experiment.

## Data Availability

The data that support the findings of this study are available from the corresponding author upon reasonable request. All related study data will be provided according to the related data management plan as open access at https://zenodo.org/.
