# Supplementary material for: The ratio of ursodeoxycholyltaurine to 7‐oxolithocholyltaurine serves as a biomarker of decreased 11β‐hydroxysteroid dehydrogenase 1 activity in mouse
Source: Br J Pharmacol. 2021 Feb 4;178(16):3309–26. doi: 10.1111/bph.15367 (PMC8359391; doi:10.1111/bph.15367)
Supplement: Supplementary file 1 — Table S1. Effect sizes of bile acid profiles measured by UHPLC‐MS/MS in plasma and liver samples were determined for mice with global Hsd11b1 knockout (11KO), liver‐specific Hsd11b1 knockout (11LKO), global H6pd knockout (H6pdKO) compared to the respective control littermates. Additionally, mice treated with the pharmacological inhibitor carbenoxolone (CBX) were compared to untreated control mice. Results represent the effect size calculated based on Cohen's d effect size (ES d) with correction of unequal sample sizes for the analysis of data from non‐parametric analysis (Mann‐Whitney U‐test) calculated as Hedges g (ES g) and corresponding confidence intervals of the effect size (± CI). NA, not analysed. Figure S1. Decreased 11β‐HSD1 bile acid product to substrate ratios in plasma of 11KO mice. 11β‐HSD1 bile acid substrates (7oxoLCA, 7oxoLC‐Tau) and products (UDCA, CDCA and their taurine conjugated forms) were measured in plasma of 11KO mice (nmol·L−1). Calculation of the product to substrate ratios attenuated the large animal‐to‐animal variations and detected the lack of 11β‐HSD1 oxoreduction activity. a) Plasma concentrations of CTRL (n = 18) and 11KO mice (n = 17) for 7oxoLCA, CDCA, UDCA and the corresponding ratios; and b) for 7oxoLCTau, CDC‐Tau, UDC‐Tau and the corresponding ratios. The results represent mean ± SEM. No outliers were excluded. Analyte concentrations defined by a S/N ≤ 3 represent the LLOD of the UHPLC‐MS/MS method. Samples yielding a concentration below LLOD were included as LLOD/2 in the calculations of a specific analyte. *P<0.05 significantly different as indicated; non‐parametric, Mann‐Whitney U‐test (two‐tailed). Unequal group sizes reflect exclusion of one plasma sample due to insufficient collection of blood sample volume. Figure S2. Decreased 11β‐HSD1 bile acid product to substrate ratios in liver tissue of 11KO mice. 11β‐ HSD1 bile acid substrates (7oxoLCA, 7oxoLC‐Tau) and products (UDCA, CDCA and their taurine conjugated forms) were me [file BPH-178-3309-s001.pdf]

Supplementary information

Supplementary Table 1

Effect sizes of bile acid profiles measured by UHPLC-MS/MS in plasma and liver samples were determined for mice with global *Hsd11b1* knockout (11KO), liver-specific *Hsd11b1* knockout (11LKO), global *H6pd* knockout (*H6pd*KO) compared to the respective control littermates. Additionally, mice treated with the pharmacological inhibitor carbenoxolone (CBX) were compared to untreated control mice. Results represent the effect size calculated based on Cohen's d effect size (ES d) with correction of unequal sample sizes for the analysis of data from non-parametric analysis (Mann-Whitney U-test) calculated as Hedges g (ES g) and corresponding confidence intervals of the effect size (± CI). NA, not analysed.

Analyte	11 KO Plasma [nmol L^{-1}]			11KO Liver [fmol mg^{-1}]			11LKO Plasma [nmol L^{-1}]			11LKO Liver [fmol mg^{-1}]			11H6pdKO Plasma [nmol L^{-1}]			H6pdKO Liver [fmol mg^{-1}]			CBX Plasma [nmol L^{-1}]			CBX Liver [fmol mg^{-1}]		
	ES (g)	CI +	CI -	ES (g)	CI +	CI -	ES (g)	CI +	CI -	ES (g)	CI +	CI -	ES (g)	CI +	CI -	ES (g)	CI +	CI -	ES (g)	CI +	CI -	ES (g)	CI +	CI -
CA	2.4	3.3	1.6	1.5	2.6	0.5	2.0	2.8	1.2	3.6	4.7	2.5	-0.9	-0.2	-1.5	-1.3	-0.6	-2.0	-0.9	0.1	-2.0	-2.3	-1.0	-3.6
CDCA	2.3	3.2	1.5	0.8	1.8	-0.2	2.6	3.5	1.7	1.8	2.6	1.0	-0.1	0.6	-0.7	-0.4	0.2	-1.1	-1.5	-0.3	-2.6	-3.8	-2.1	-5.5
DCA	2.7	3.6	1.8	1.1	2.0	0.1	0.5	1.2	-0.2	1.0	1.7	0.3	-2.4	-1.6	-3.3	-2.8	-1.9	-3.7	-2.2	-0.9	-3.5	-6.8	-4.1	-9.4
7oxoDCA	2.7	3.6	1.8	1.8	2.9	0.7	2.0	2.8	1.2	4.4	5.7	3.2	0.3	0.9	-0.4	0.2	0.8	-0.5	0.2	1.2	-0.8	1.1	2.2	0.0
HDCA	4.4	5.6	3.1	3.0	4.3	1.6	0.9	1.6	0.1	2.8	3.8	1.9	-0.2	0.4	-0.8	-1.3	-0.6	-2.0	-1.8	-0.6	-3.0	-4.1	-2.3	-5.9
αMCA	3.0	4.0	2.0	2.4	3.6	1.2	1.8	2.6	1.0	4.3	5.6	3.1	0.3	0.9	-0.4	-0.6	0.1	-1.2	-1.4	-0.3	-2.6	-4.2	-2.4	-6.0
βMCA	0.9	1.6	0.2	-3.4	-2.0	-4.8	-4.8	-3.5	-6.2	-0.5	0.2	-1.2	-2.0	-1.2	-2.8	-4.5	-3.3	-5.7	-0.9	0.2	-2.0	2.6	3.9	1.2
ωMCA	2.0	2.9	1.2	0.1	1.0	-0.8	0.1	0.8	-0.5	3.2	4.2	2.1	-1.4	-0.7	-2.1	-3.5	-2.5	-4.6	-1.9	-0.7	-3.1	2.1	3.4	0.9
UDCA	2.6	3.5	1.7	0.9	1.9	-0.1	0.1	0.8	-0.6	3.5	4.6	2.4	-1.6	-0.9	-2.4	-3.5	-2.5	-4.5	-2.0	-0.7	-3.2	-7.1	-4.4	-9.9
7oxoLCA	2.7	3.6	1.8	2.8	4.1	1.5	2.6	3.5	1.6	2.8	3.7	1.8	3.5	4.5	2.5	3.0	3.9	2.1	NA			NA		
αMCA/βMCA	14.3	17.7	10.9	30.7	40.8	20.6	24.0	29.8	18.2	49.3	61.2	37.4	54.6	66.9	42.3	42.2	51.8	32.7	1.3	2.4	0.2	-21.1	-13.5	-28.8
UDCA/7oxoLCA	-8.4	-6.3	-10.5	-11.6	-7.7	-15.6	-17.0	-12.9	-21.2	5.2	6.6	3.7	-16.6	-12.8	-20.4	-34.2	-26.5	-41.9	NA			NA		
CDCA/7oxoLCA	-8.1	-6.1	-10.1	-13.5	-9.0	-18.0	-8.3	-6.2	-10.4	-7.7	-5.7	-9.7	-7.6	-5.8	-9.5	-17.2	-13.2	-21.1	NA			NA		
C-Tau	9.1	11.3	6.9	6.1	8.3	3.9	-2.8	-1.9	-3.8	2.6	3.6	1.7	3.8	4.9	2.8	-8.4	-6.4	-10.4	1.6	2.7	0.4	-4.9	-2.9	-6.9
CDC-Tau	8.9	11.1	6.7	6.9	9.4	4.5	0.2	0.9	-0.5	3.2	4.3	2.2	4.8	6.0	3.5	1.4	2.1	0.7	-1.3	-0.2	-2.4	-6.5	-4.0	-9.1
DC-Tau	8.7	10.8	6.5	4.0	5.7	2.4	3.8	4.9	2.6	-5.7	-4.2	-7.3	3.3	4.3	2.3	-6.1	-4.6	-7.6	-2.0	-0.8	-3.3	-21.9	-14.0	-29.8
LC-Tau	9.6	12.0	7.3	6.1	8.2	3.9	-110.3	-83.7	-136.9	-4.8	-3.5	-6.2	5.6	7.0	4.2	3.2	4.1	2.2	-5.7	-3.4	-8.0	-23.8	-15.2	-32.3
αMCA-Tau + βMCA-Tau	8.4	10.5	6.3	0.6	1.5	-0.4	3.8	4.9	2.6	-0.7	0.0	-1.4	-2.8	-1.9	-3.7	-14.3	-11.0	-17.6	-1.2	-0.1	-2.3	2.9	4.4	1.5
ωMCA-Tau	33.2	41.0	25.4	-8.6	-5.6	-11.5	-3.8	-2.6	-4.9	-4.8	-3.5	-6.2	1.6	2.3	0.8	-14.0	-10.8	-17.2	4.3	6.2	2.5	3.9	5.7	2.2
UDC-Tau	7.2	9.0	5.4	-25.5	-17.1	-33.9	-10.8	-8.1	-13.5	-19.9	-15.0	-24.7	1.9	2.6	1.1	-15.8	-12.2	-19.4	0.5	1.6	-0.5	-26.9	-17.2	-36.5
7oxoLC-Tau	11.8	14.6	8.9	12.4	16.5	8.2	12.6	15.8	9.5	21.1	26.2	15.9	8.6	10.7	6.6	22.8	28.0	17.6	1.9	3.1	0.7	-1.4	-0.3	-2.5
UDC-Tau/7oxoLC-Tau	-46.7	-35.7	-57.6	-32.7	-22.0	-43.5	-8.0	-5.9	-10.0	-41.2	-31.3	-51.2	-36.8	-28.5	-45.1	-61.3	-47.5	-75.0	-14.9	-9.5	-20.3	-22.2	-14.2	-30.2
CDC-Tau/7oxoLC-Tau	-13.4	-10.2	-16.5	-30.5	-20.5	-40.4	-7.6	-5.7	-9.6	-26.2	-19.8	-32.5	-32.2	-24.9	-39.4	-47.8	-37.0	-58.5	-9.0	-5.6	-12.4	-5.3	-3.1	-7.4
Sum primary BA	14.6	18.0	11.1	4.7	6.5	2.9	8.6	10.8	6.4	16.3	20.3	12.3	-7.2	-5.4	-8.9	-16.3	-12.6	-20.0	-3.9	-2.2	-5.6	7.7	10.6	4.8
Sum primary BA-	8.9	11.1	6.7	4.0	5.6	2.4	0.1	0.7	-0.6	1.3	2.1	0.6	3.5	4.5	2.5	-10.3	-7.9	-12.7	1.0	2.1	-0.1	-2.4	-1.0	-3.7

Supplementary Figure 1

Decreased 11β-HSD1 bile acid product to substrate ratios in plasma of 11KO mice. 11β-HSD1 bile acid substrates (7oxoLCA, 7oxoLC-Tau) and products (UDCA, CDCA and their taurine conjugated forms) were measured in plasma of 11KO mice (nmol·L^{-1}). Calculation of the product to substrate ratios attenuated the large animal-to-animal variations and detected the lack of 11β-HSD1 oxoreduction activity. a) Plasma concentrations of CTRL (n = 18) and 11KO mice (n = 17) for 7oxoLCA, CDCA, UDCA and the corresponding ratios; and b) for 7oxoLC-Tau, CDC-Tau, UDC-Tau and the corresponding ratios. The results represent mean ± SEM. No outliers were excluded. Analyte concentrations defined by a S/N ≤ 3 represent the LLOD of the UHPLC-MS/MS method. Samples yielding a concentration below LLOD were included as LLOD/2 in the calculations of a specific analyte. *P<0.05 significantly different as indicated; non-parametric, Mann-Whitney U-test (two-tailed). Unequal group sizes reflect exclusion of one plasma sample due to insufficient collection of blood sample volume.

Supplementary Figure 2

Decreased 11β-HSD1 bile acid product to substrate ratios in liver tissue of 11KO mice. 11β-HSD1 bile acid substrates (7oxoLCA, 7oxoLC-Tau) and products (UDCA, CDCA and their taurine conjugated forms) were measured in liver tissue of 11KO mice (fmol·mg^{-1}). Calculation of the product to substrate ratios attenuated the large animal-to-animal variations and detected the lack of 11β-HSD1 oxoreduction activity. a) Liver tissue concentrations of CTRL (n = 9) and 11KO mice (n = 9) for 7oxoLCA, CDCA, UDCA and the corresponding ratios; and b) for 7oxoLC-Tau, CDC-Tau, UDC-Tau and the corresponding ratios. The results represent mean ± SEM. No outliers were excluded. Analyte concentrations defined by a S/N ≤ 3 represent the LLOD of the UHPLC-MS/MS method. Samples yielding a concentration below LLOD were included as LLOD/2 in the calculations of a specific analyte. *P<0.05 significantly different as indicated; non-parametric, Mann-Whitney U-test (two-tailed). Unequal group sizes reflect the availability of only nine livers due to the use of nine randomly assigned livers for gene expression analyses in a previous study.

Supplementary Figure 3

Decreased 11β-HSD1 bile acid product to substrate ratios in plasma of 11LKO mice. 11β-HSD1 bile acid substrates (7oxoLCA, 7oxoLC-Tau) and products (UDCA, CDCA and their taurine conjugated forms) were measured in plasma of 11LKO mice (nmol·L^{-1}). Calculation of the product to substrate ratios attenuated the large animal-to-animal variations and detected the

decreased 11β-HSD1 oxoreduction activity. a) Plasma concentrations for 7oxoLCA, CDCA, UDCA and the corresponding ratios, and b) for 7oxoLC-Tau, CDC-Tau, UDC-Tau and the corresponding ratios of CTRL (n = 17) and 11LKO mice (n = 16). The results represent mean ± SEM. No outliers were excluded. Analyte concentrations defined by a S/N ≤ 3 represent the LLOD of the UHPLC-MS/MS method. Samples yielding a concentration below LLOD were included as LLOD/2 in the calculations of a specific analyte. *P<0.05 significantly different as indicated; non-parametric, Mann-Whitney U-test (two-tailed). Unequal group sizes reflect exclusion of one 11LKO animal due to unexpected health issues prior to reaching the age for the experiment.

Supplementary Figure 4

Decreased 11β-HSD1 bile acid product to substrate ratios in liver tissue of 11LKO mice. 11β-HSD1 bile acid substrates (7oxoLCA, 7oxoLC-Tau) and products (UDCA, CDCA and their taurine conjugated forms) were measured in liver tissue of 11LKO mice ($fmol \cdot mg^{-1}$). Calculation of the product to substrate ratios attenuated the large animal-to-animal variations and detected the decreased 11β-HSD1 oxoreduction activity. a) Liver tissue concentrations for 7oxoLCA, CDCA, UDCA and the corresponding ratios, and b) for 7oxoLC-Tau, CDC-Tau, UDC-Tau and the corresponding ratios of CTRL (n = 17) and 11LKO mice (n = 16). The results represent mean ± SEM. No outliers were excluded. Analyte concentrations defined by a S/N ≤ 3 represent the LLOD of the UHPLC-MS/MS method. Samples yielding a concentration below LLOD were included as LLOD/2 in the calculations of a specific analyte. *P<0.05 significantly different as indicated; non-parametric, Mann-Whitney U-test (two-tailed). Unequal group sizes reflect exclusion of one 11LKO animal due to unexpected health issues prior to reaching the age for the experiment.

Supplementary Figure 5

Decreased 11β-HSD1 oxoreduction activity in white adipose tissue of *H6pd*KO mice. a) Estimation of the conversion of cortisone to cortisol, and b) of cortisol to cortisone measured *ex vivo* in mouse white adipose tissue (CTRL n = 7; *H6pd*KO n = 7). Results represent mean ± SEM, *P<0.05 significantly different as indicated; non-parametric, Mann-Whitney U-test (two-tailed).

Supplementary Figure 6:

Decreased 11β-HSD1 bile acid product to substrate ratios in plasma of *H6pd*KO mice. 11β-HSD1 bile acid substrates (7oxoLCA, 7oxoLC-Tau) and products (UDCA, CDCA and their taurine conjugated forms) were measured in plasma of *H6pd*KO mice. Calculation of the product to substrate ratios attenuated the large animal-to-animal variations and detected the decreased 11β-HSD1 oxoreduction activity. a) Plasma concentrations (nmol·L^{-1}) for 7oxoLCA, CDCA, UDCA and the corresponding ratios, and b) for 7oxoLC-Tau, CDC-Tau, UDC-Tau and the corresponding ratios of CTRL (n = 20) and *H6pd*KO mice (n = 18). The results represent mean ± SEM. No outliers were excluded. Analyte concentrations defined by a S/N ≤ 3 represent the LLOD of the UHPLC-MS/MS method. Samples yielding a concentration below LLOD were included as LLOD/2 in the calculations of a specific analyte. *P<0.05 significantly different as indicated; non-parametric, Mann-Whitney U-test (two-tailed). Unequal group sizes reflect exclusion of two *H6pd*KO animals from further analysis due to the occurrence of liver cysts.

Supplementary Figure 7

Decreased 11β-HSD1 bile acid product to substrate ratios in liver tissue of *H6pd*KO mice. 11β-HSD1 bile acid substrates (7oxoLCA, 7oxoLC-Tau) and products (UDCA, CDCA and their taurine conjugated forms) were measured in liver tissue of *H6pd*KO mice. Calculation of the product to substrate ratios attenuated the large animal-to-animal variations and detected the decreased 11β-HSD1 oxoreduction activity. a) Liver tissue concentrations (fmol·mg^{-1}) for 7oxoLCA, CDCA, UDCA and the corresponding ratios, and b) for 7oxoLC-Tau, CDC-Tau, UDC-Tau and the corresponding ratios of CTRL (n = 20) and *H6pd*KO mice (n = 18). The results represent mean ± SEM. No outliers were excluded. Analyte concentrations defined by a S/N ≤ 3 represent the LLOD of the UHPLC-MS/MS method. Samples yielding a concentration below LLOD were included as LLOD/2 in the calculations of a specific analyte. *P<0.05 significantly different as indicated; non-parametric, Mann-Whitney U-test (two-tailed). Unequal group sizes reflect exclusion of two *H6pd*KO animals from further analysis due to the occurrence of liver cysts.

Supplementary Figure 8

Metabolism of 7oxoLCA to UDCA and CDCA by murine 11β-HSD1 and inhibition by CBX. HEK-293 cells expressing murine 11β-HSD1 (MO1F) were incubated with 400 nmol L^{-1} 7oxoLCA in the absence and presence of 5 μmol L^{-1} of CBX, followed by quantification of

substrate and products. a) 7oxoLCA concentration [nmol L^{-1}] after 4 h and 24 h of incubation. b) CDCA formation represented as concentration [nmol L^{-1}] after 4 h and 24h of incubation. c) UDCA formation represented as concentration [nmol L^{-1}] after 4 h and 24 h of incubation. d) Representative western blot analysis of protein levels of 11β-HSD1 in untransfected HEK-293 cells and in MO1F cells. ACTB served as loading control. The results represent mean ± SD, n=5. *P<0.05 significantly different as indicated; non-parametric, Mann-Whitney U-test (two-tailed).

Supplementary Figure 9

Decreased 11β-HSD1 bile acid product to substrate ratios in plasma of mice treated with the inhibitor carbenoxolone (CBX). 11β-HSD1 bile acid substrates (7oxoLCA, 7oxoLC-Tau) and products (UDCA, CDCA and their taurine conjugated forms) were measured in plasma of control mice either treated with the pharmacologic 11β-HSD1 inhibitor CBX (100 mg kg^{-1} d^{-1}, *i.p.*) or PBS (CTRL) (CTRL n = 8; CBX n = 7). Calculation of the product/substrate ratios attenuated the large animal-to-animal variations and detected the diminished 11β-HSD1 oxoreduction activity. a) Plasma concentrations (nmol·L^{-1}) of 7oxoLCA, CDCA, UDCA and the corresponding ratios. Some values were below LLOD. b) Plasma concentrations (nmol·L^{-1}) of 7oxoLC-Tau, CDC-Tau, UDC-Tau and the corresponding ratios. The results represent mean ± SEM. No outliers were excluded. Analyte concentrations defined by a S/N of ≤ 3 represent the LLOD of the UHPLC-MS/MS method. Samples yielding a concentration below LLOD were included as LLOD/2 in the calculations of a specific analyte. *P<0.05 significantly different as indicated; non-parametric, Mann-Whitney U-test (two-tailed). Unequal group sizes reflect exclusion of one animal of the CTRL group due to unexpected health issues prior to the experiment and exclusion of two plasma samples of the CBX group due to insufficient collection of blood sample volume.

Supplementary Figure 10

Decreased 11β-HSD1 bile acid product to substrate ratios in liver tissue of mice treated with the inhibitor carbenoxolone (CBX). 11β-HSD1 bile acid substrates (7oxoLCA, 7oxoLC-Tau) and products (UDCA, CDCA and their taurine conjugated forms) were measured in liver tissue of control mice either treated with the pharmacologic 11β-HSD1 CBX (100 mg kg^{-1} d^{-1}, *i.p.*) or PBS (CTRL) (CTRL n = 8; WT CBX n = 9). Calculation of the product/substrate ratios attenuated the large animal-to-animal variations and detected the diminished 11β-HSD1 oxoreduction activity. a) Liver tissue concentrations (fmol·mg^{-1}) of 7oxoLCA, CDCA, UDCA

and the corresponding ratios. Some values were below LLOD. b) Liver tissue concentrations (fmol·mg^{-1}) of 7oxoLC-Tau, CDC-Tau, UDC-Tau and the corresponding ratios. The results represent mean ± SEM. No outliers were excluded. Analyte concentrations defined by a S/N ≤ 3 represent the LLOD of the UHPLC-MS/MS method. Samples yielding a concentration below LLOD were included as LLOD/2 in the calculations of a specific analyte. *$P<0.05$ significantly different as indicated; non-parametric, Mann-Whitney U-test (two-tailed). Unequal group sizes reflect exclusion of one animal of the CTRL group due to unexpected health issues prior to the experiment.

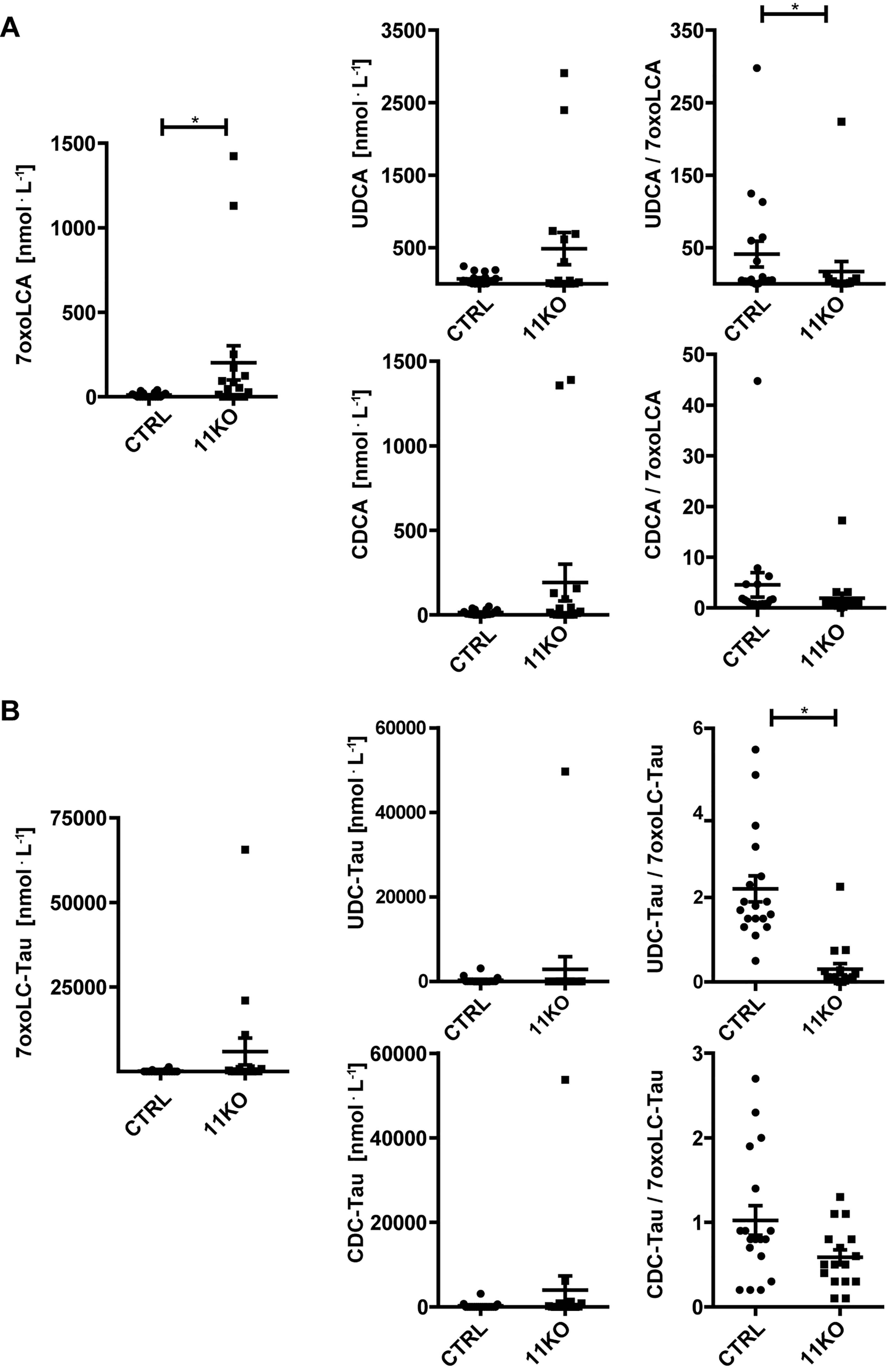
A
7oxoLCA [nmol · L-1]
CTRL
11KO
UDCA [nmol · L-1]
UDCA / 7oxoLCA
CDCA [nmol · L-1]
CDCA / 7oxoLCA
B
7oxoLC-Tau [nmol · L-1]
UDC-Tau [nmol · L-1]
UDC-Tau / 7oxoLC-Tau
CDC-Tau [nmol · L-1]
CDC-Tau / 7oxoLC-Tau

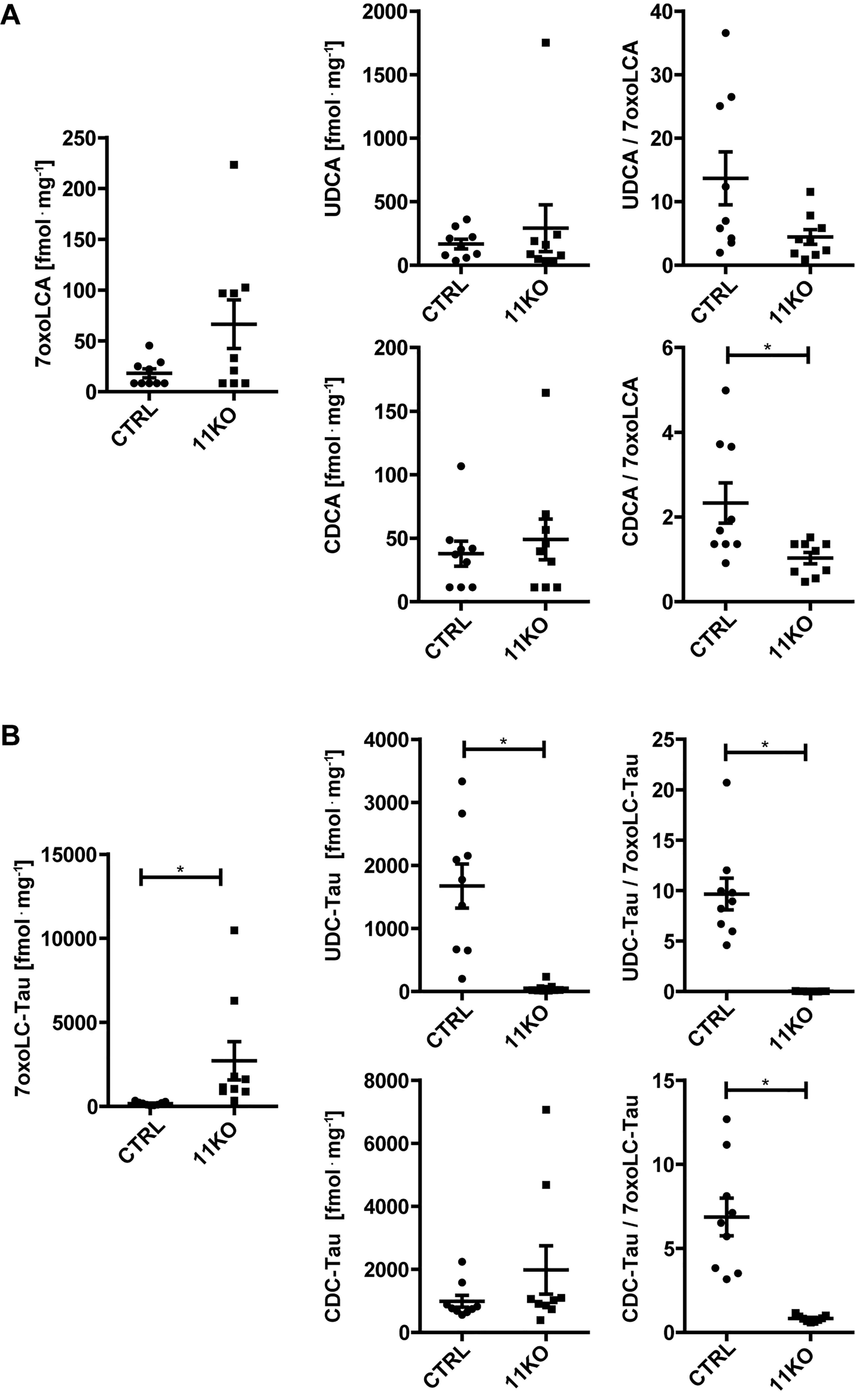
A
7oxoLCA [fmol · mg-1]
UDCA [fmol · mg-1]
UDCA / 7oxoLCA
CDCA [fmol · mg-1]
CDCA / 7oxoLCA
CTRL
11KO
B
7oxoLC-Tau [fmol · mg-1]
UDC-Tau [fmol · mg-1]
UDC-Tau / 7oxoLC-Tau
CDC-Tau [fmol · mg-1]
CDC-Tau / 7oxoLC-Tau
*

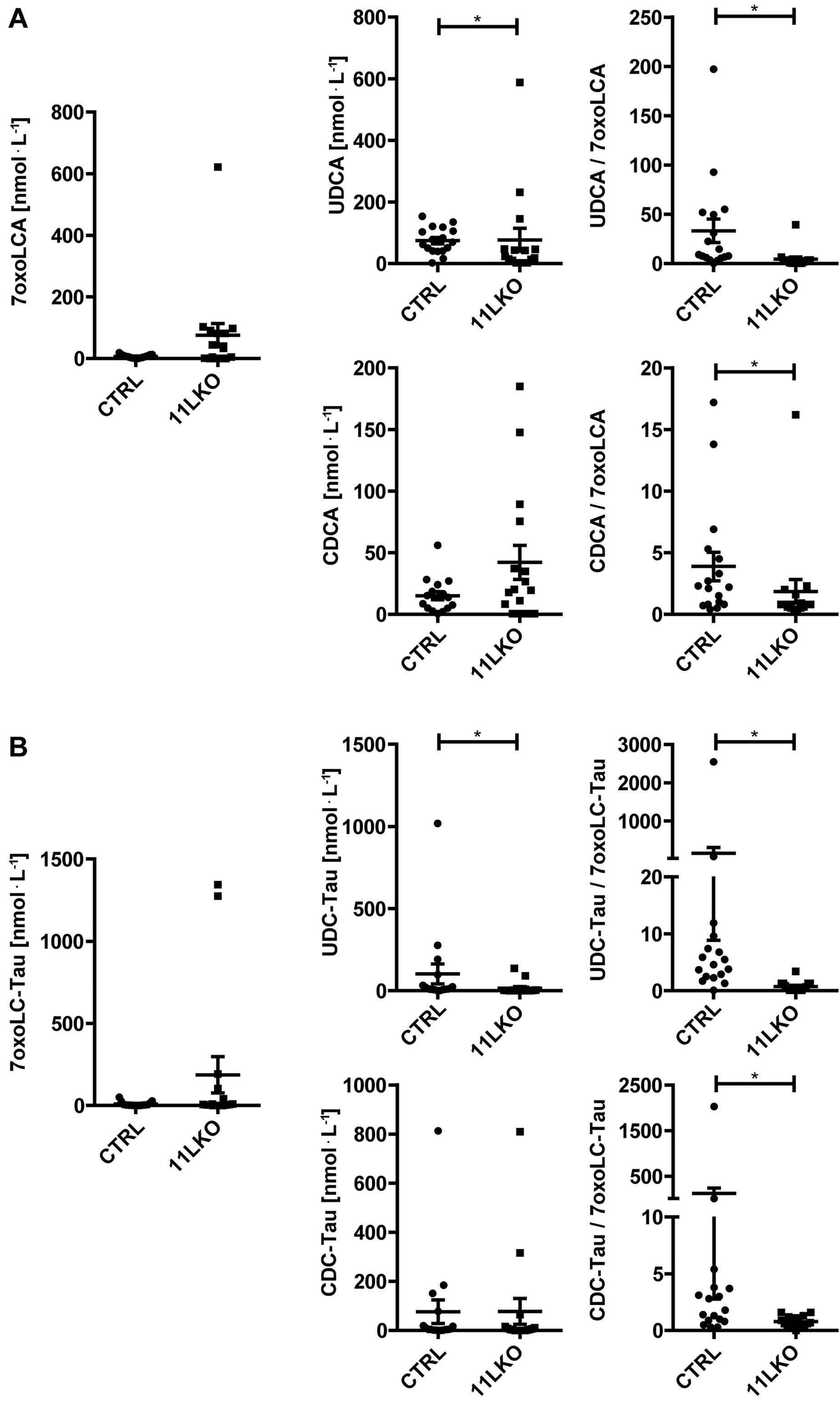
A
7oxoLCA [nmol · L-1]
UDCA [nmol · L-1]
UDCA / 7oxoLCA
CDCA [nmol · L-1]
CDCA / 7oxoLCA
B
7oxoLC-Tau [nmol · L-1]
UDC-Tau [nmol · L-1]
UDC-Tau / 7oxoLC-Tau
CDC-Tau [nmol · L-1]
CDC-Tau / 7oxoLC-Tau
CTRL
11LKO
*

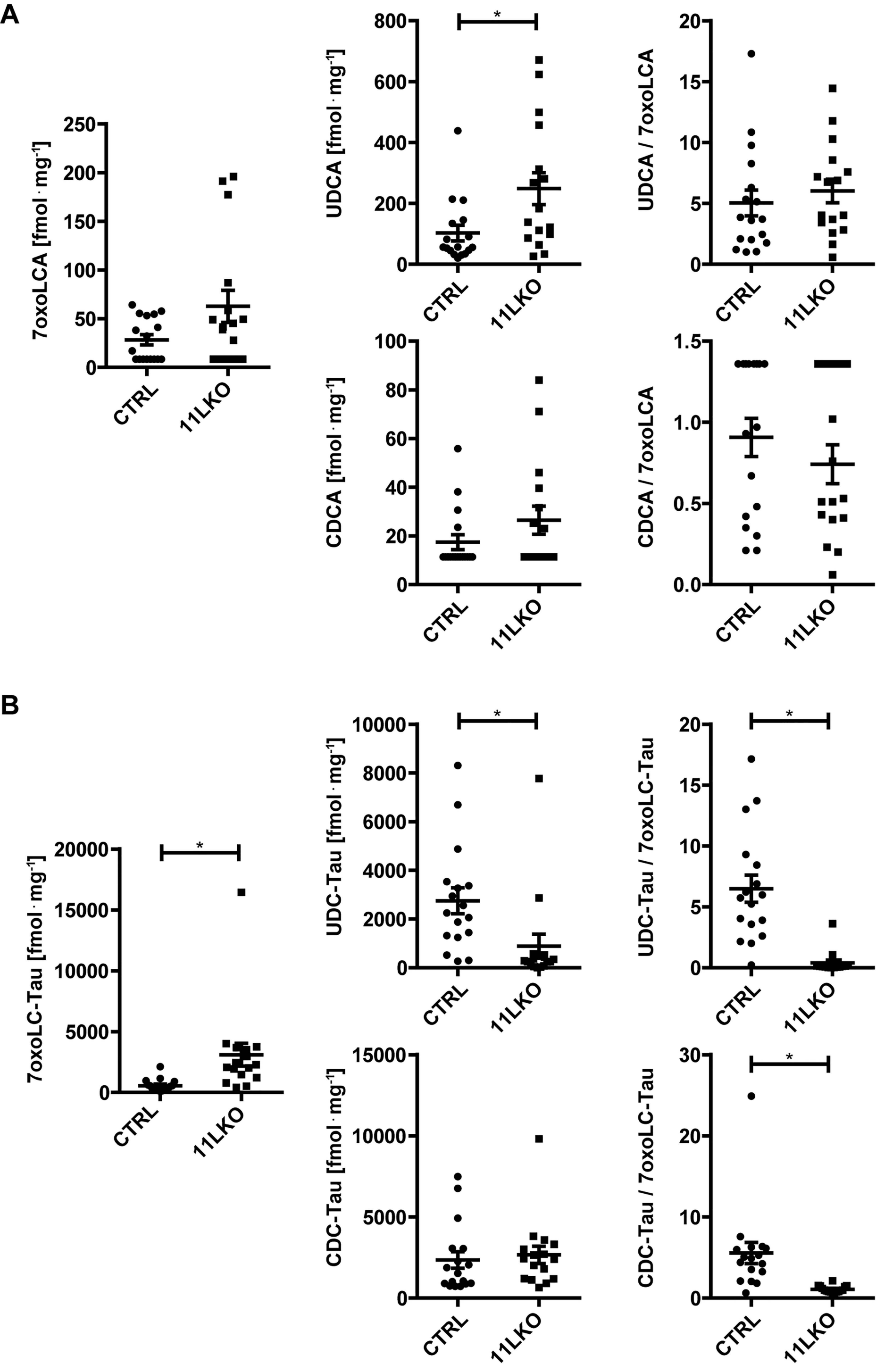

A
7oxoLCA [fmol·mg-1]
UDCA [fmol·mg-1]
UDCA / 7oxoLCA
CDCA [fmol·mg-1]
CDCA / 7oxoLCA
B
7oxoLC-Tau [fmol·mg-1]
UDC-Tau [fmol·mg-1]
UDC-Tau / 7oxoLC-Tau
CDC-Tau [fmol·mg-1]
CDC-Tau / 7oxoLC-Tau
CTRL
11LKO
*

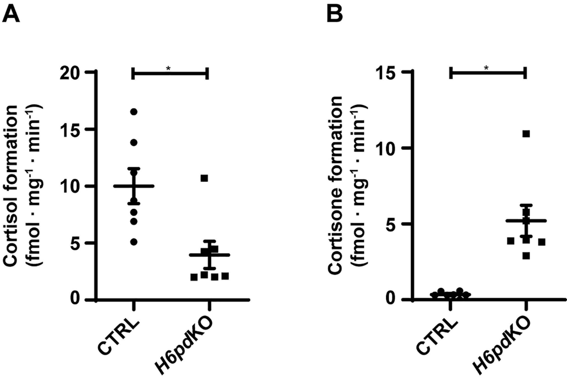
A
B
Cortisol formation
(fmol · mg-1 · min-1)
20
15
10
5
0
*
CTRL
H6pdKO
Cortisone formation
(fmol · mg-1 · min-1)
15
10
5
0
*
CTRL
H6pdKO

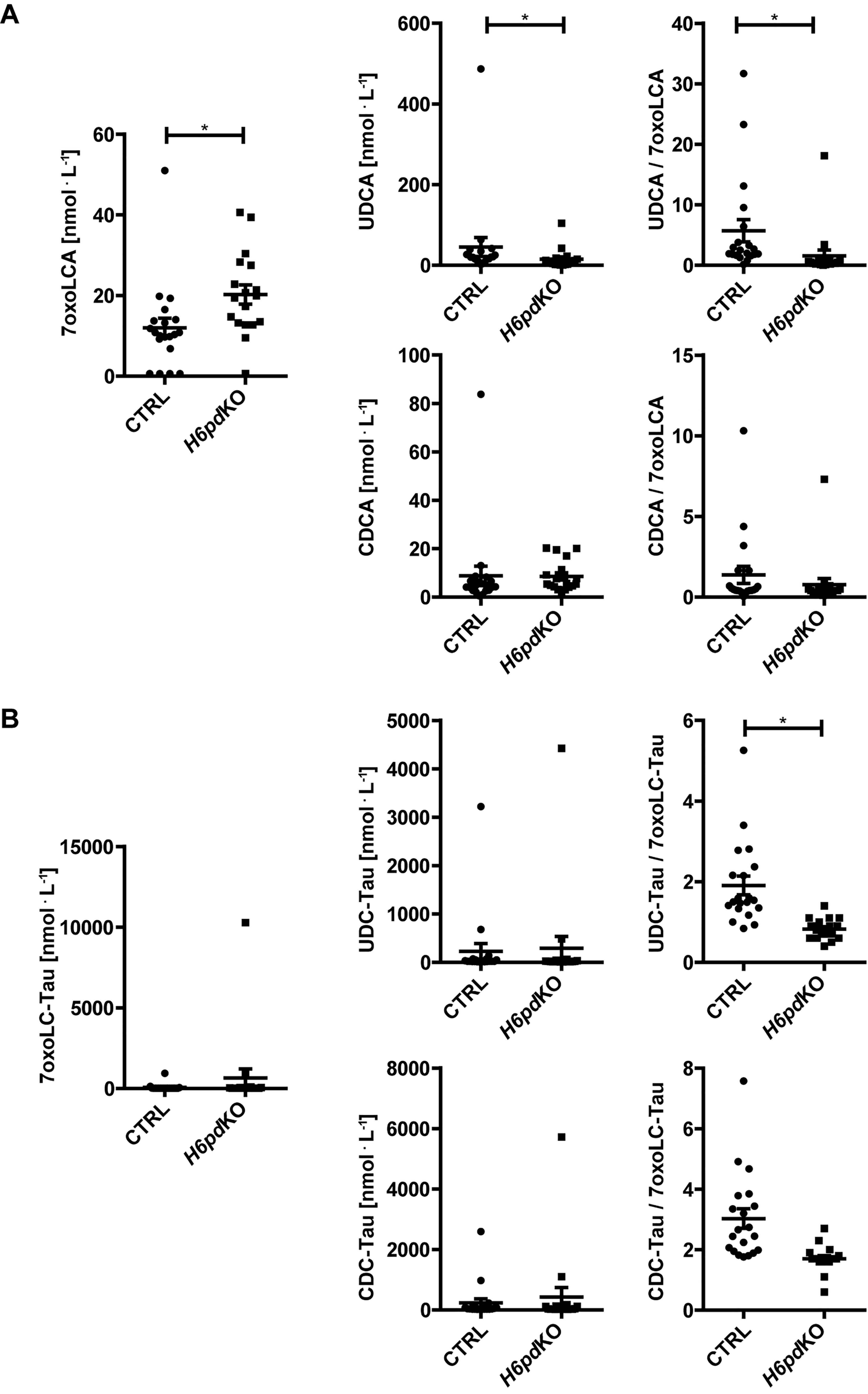

A
7oxoLCA [nmol · L-1]
UDCA [nmol · L-1]
UDCA / 7oxoLCA
CDCA [nmol · L-1]
CDCA / 7oxoLCA
B
7oxoLC-Tau [nmol · L-1]
UDC-Tau [nmol · L-1]
UDC-Tau / 7oxoLC-Tau
CDC-Tau [nmol · L-1]
CDC-Tau / 7oxoLC-Tau
CTRL
H6pdKO
*

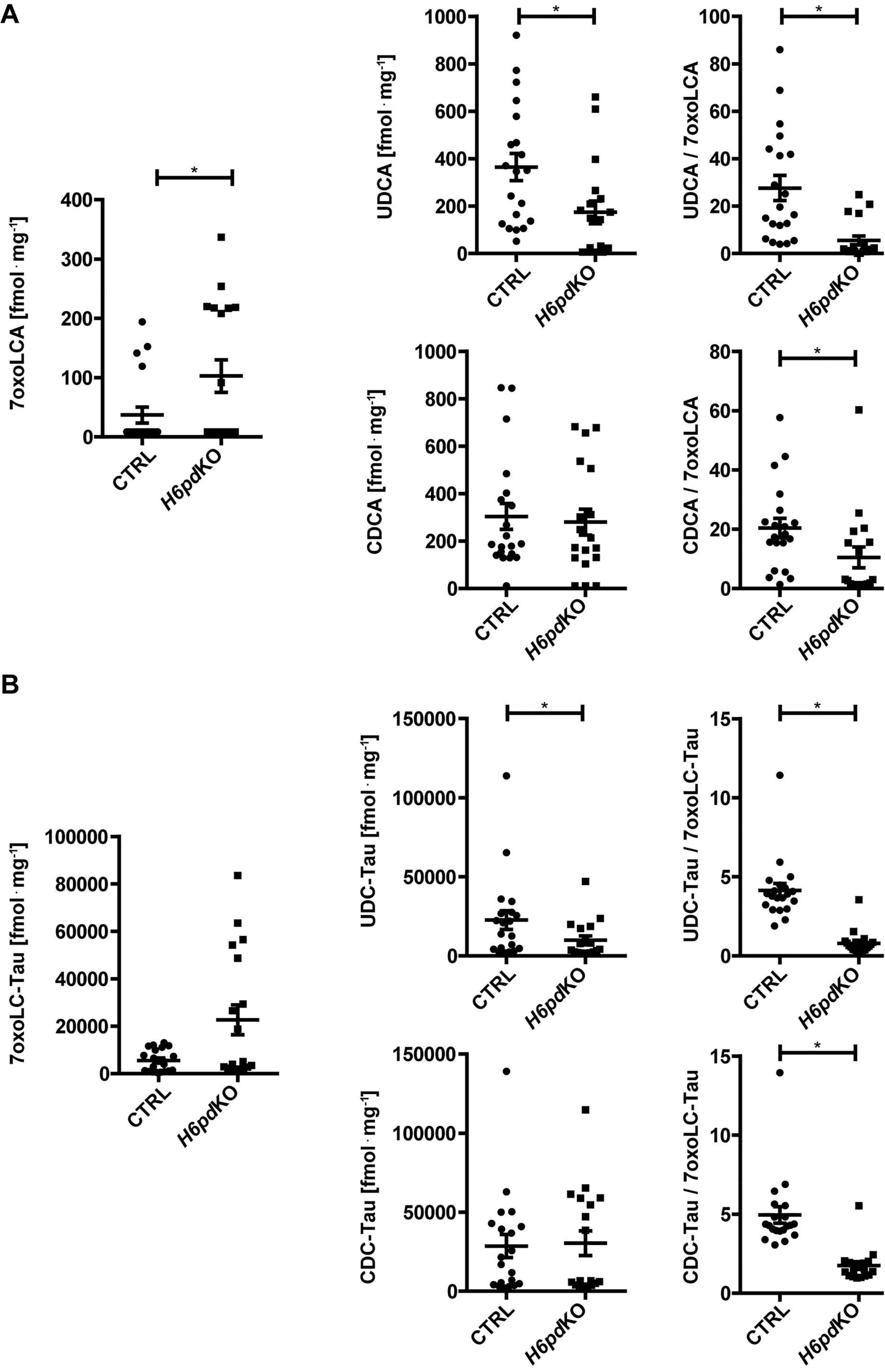
A
B
7oxoLCA [fmol · mg-1]
UDCA [fmol · mg-1]
UDCA / 7oxoLCA
CDCA [fmol · mg-1]
CDCA / 7oxoLCA
7oxoLC-Tau [fmol · mg-1]
UDC-Tau [fmol · mg-1]
UDC-Tau / 7oxoLC-Tau
CDC-Tau [fmol · mg-1]
CDC-Tau / 7oxoLC-Tau
CTRL
H6pdKO
*

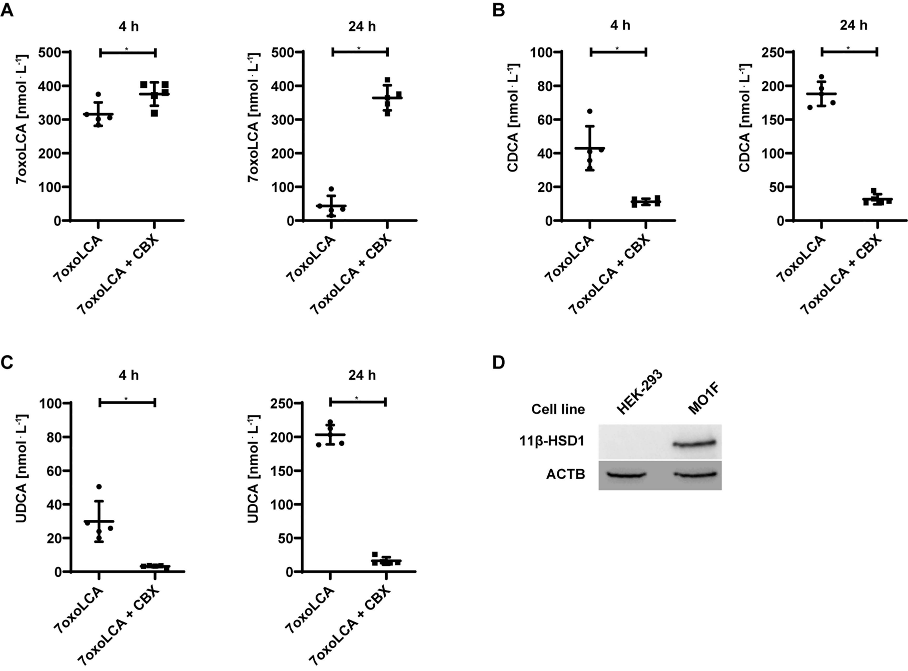

A
4 h
24 h
7oxoLCA [nmol·L-1]
7oxoLCA
7oxoLCA + CBX
B
CDCA [nmol·L-1]
C
UDCA [nmol·L-1]
D
Cell line
HEK-293
MO1F
11β-HSD1
ACTB

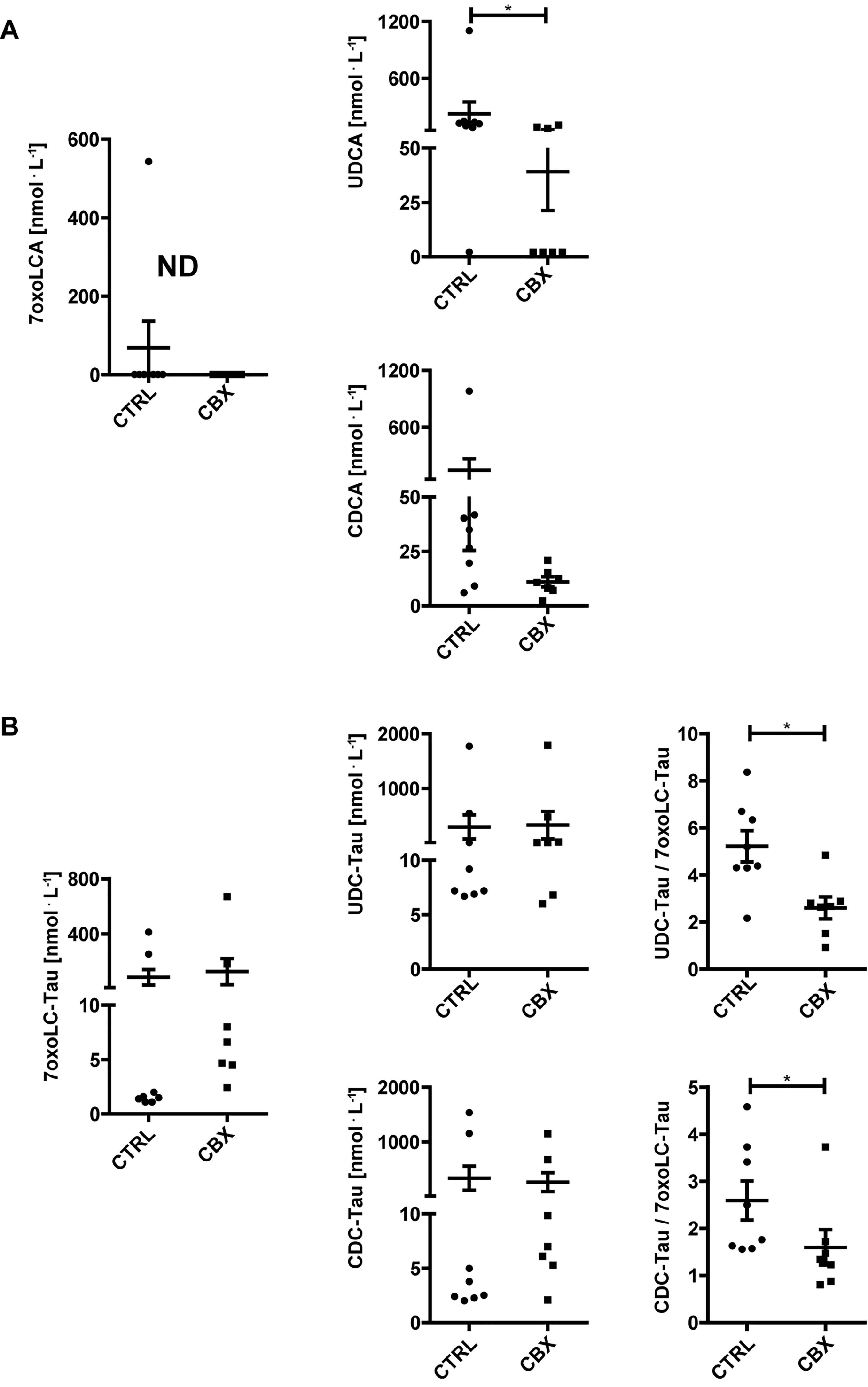
A
7oxoLCA [nmol · L-1]
600
400
200
0
ND
CTRL
CBX
UDCA [nmol · L-1]
1200
600
50
25
0
*
CTRL
CBX
CDCA [nmol · L-1]
1200
600
50
25
0
CTRL
CBX
B
7oxoLC-Tau [nmol · L-1]
800
400
10
5
0
CTRL
CBX
UDC-Tau [nmol · L-1]
2000
1000
10
5
0
CTRL
CBX
UDC-Tau / 7oxoLC-Tau
10
8
6
4
2
0
*
CTRL
CBX
CDC-Tau [nmol · L-1]
2000
1000
10
5
0
CTRL
CBX
CDC-Tau / 7oxoLC-Tau
5
4
3
2
1
0
*
CTRL
CBX

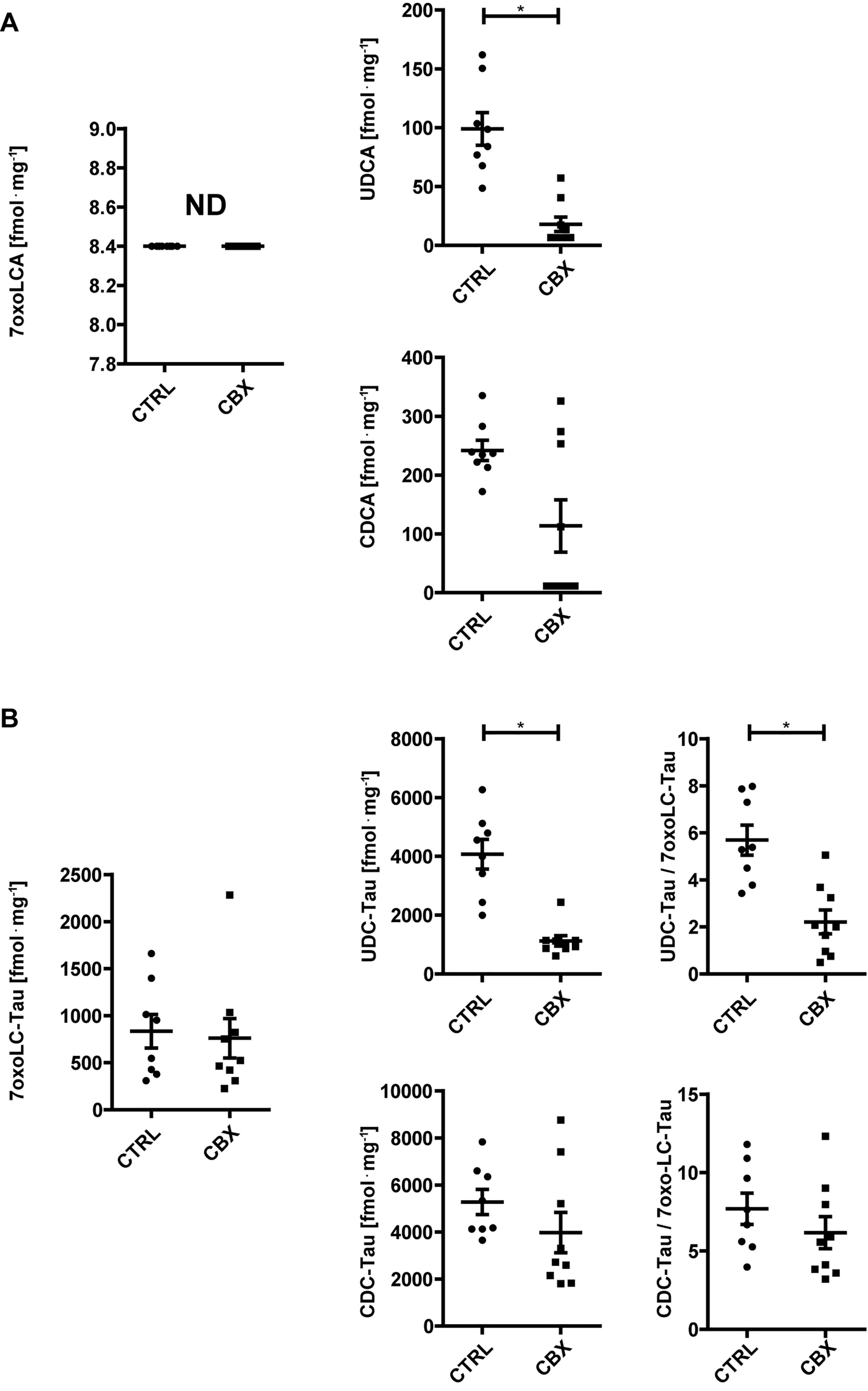
A
7oxoLCA [fmol·mg-1]
9.0
8.8
8.6
8.4
8.2
8.0
7.8
ND
CTRL
CBX
UDCA [fmol·mg-1]
200
150
100
50
0
*
CDCA [fmol·mg-1]
400
300
200
100
0
B
7oxoLC-Tau [fmol·mg-1]
2500
2000
1500
1000
500
0
UDC-Tau [fmol·mg-1]
8000
6000
4000
2000
UDC-Tau / 7oxoLC-Tau
10
8
6
4
2
CDC-Tau [fmol·mg-1]
10000
CDC-Tau / 7oxo-LC-Tau
15
5
